# miRNAs, lncRNAs, circRNAs and piRNAs in Nonalcoholic Fatty Liver Disease: Past, Present and Future

**DOI:** 10.3390/ijms262110402

**Published:** 2025-10-26

**Authors:** Roxana Liana Lucaciu, Olga Hilda Orasan, Adriana Corina Hangan, Mihaela Iancu, Angela Cozma, Sorina Cezara Coste, Sidonia Gog-Bogdan, Bogdan Sevastre, Lucia Maria Procopciuc

**Affiliations:** 1 Department of Pharmaceutical Biochemistry and Clinical Laboratory, Faculty of Pharmacy, “Iuliu Hațieganu” University of Medicine and Pharmacy, 400012 Cluj-Napoca, Romania; liana.lucaciu@umfcluj.ro; 2 4th Department of Internal Medicine, Faculty of Medicine, “Iuliu Hațieganu” University of Medicine and Pharmacy, 400012 Cluj-Napoca, Romania; hilda.orasan@umfcluj.ro (O.H.O.); angelacozma@umfcluj.ro (A.C.); secara.sorina@yahoo.com (S.C.C.); 3 Department of Inorganic Chemistry, Faculty of Pharmacy, “Iuliu Hațieganu” University of Medicine and Pharmacy, 400012 Cluj-Napoca, Romania; adriana.hangan@umfcluj.ro; 4 Medical Informatics and Biostatistics, Faculty of Nursing and Health Science, “Iuliu Hațieganu” University of Medicine and Pharmacy, 400012 Cluj-Napoca, Romania; miancu@umfcluj.ro; 5 Department of Sugery and ATI, Faculty of Veterinary Medicine, University of Agricultural Sciences and Veterinary Medicine, 400372 Cluj-Napoca, Romania; 6 Department of Paraclinics, University of Agricultural Sciences and Veterinary Medicine, 400372 Cluj-Napoca, Romania; sevastre.bogdan@usamvcluj.ro; 7 Medical Biochemistry, Department of Molecular Sciences, Faculty of Medicine, “Iuliu Hațieganu” University of Medicine and Pharmacy, 400349 Cluj-Napoca, Romania; lprocopciuc@umfcluj.ro

**Keywords:** NAFLD, ncRNAs, miRNAs, lncRNAs, circRNAs, PiRNAs

## Abstract

Nowadays, nonalcoholic fatty liver disease (NAFLD) represents the most common cause of chronic liver disorder worldwide. From the clinical point of view, it evolves from steatosis to nonalcoholic steatohepatitis, which can lead to cirrhosis and finally to hepatocellular carcinoma. The mechanisms involved in its progression to more pathological stages and NAFLD pathogenesis are not completely understood. The research concerning NAFLD has become urgent and important because the age of NAFLD diagnosis is progressively decreasing, and its relationship with cancer risk is already well known. Because NAFLD ultimately leads to disability and imposes a major socioeconomic burden, timely diagnosis and effective treatment of NAFLD is particularly important. In the development of NAFLD, noncoding RNAs (ncRNAs) represented by microRNAs, long noncoding RNAs, circular RNAs, and piRNAs are epigenetic factors that play important regulatory roles. In the current review, we present updated information regarding the role of miRNAs, lncRNAs, circRNAs, and piRNAs, aiming to develop a good understanding of their regulatory functions in hepatic metabolism and concerning their potential use as biomarkers for early NAFLD/NASH diagnosis and as therapeutic targets.

## 1. Introduction

Nowadays, nonalcoholic fatty liver disease (NAFLD) represents the most common cause of chronic liver disorder worldwide. From the clinical point of view, it evolves from steatosis to nonalcoholic steatohepatitis, which can lead to cirrhosis and finally to hepatocellular carcinoma (HCC) [1]. NAFLD is characterized by the accumulation of fat within hepatocytes, primarily due to increased triglyceride (TG) biosynthesis, driven by elevated levels of free fatty acids (FFAs) and dysregulated de novo lipogenesis. The excess FFAs contribute to oxidative stress by promoting the production of reactive oxygen and nitrogen species. As the disease progresses to nonalcoholic steatohepatitis (NASH), typical features include lobular inflammation and hepatocellular ballooning. The inflammatory environment further activates hepatic stellate cells (HSCs), which differentiate into myofibroblasts, ultimately leading to liver fibrosis [2].

Under normal physiological conditions, the liver and adipose tissue work in close coordination to sustain overall energy balance. The liver regulates carbohydrate and lipid metabolism through pathways like de novo lipogenesis, β-oxidation, and very low-density lipoprotein (VLDL) secretion. Typically, fatty acids (FAs) are produced from acetyl-CoA via acetyl-CoA carboxylase (ACC) and fatty acid synthase (FAS) [3]. Meanwhile, mitochondrial β-oxidation, under the control of peroxisome proliferator-activated receptor-α (PPARα), generates energy and helps prevent lipid accumulation [4]. Triglycerides are assembled and secreted as VLDL particles to prevent fat buildup inside cells [5]. Meanwhile, adipose tissue stores excess energy as TG and releases FFAs during fasting, along with secreting adipokines like leptin and adiponectin, which help regulate liver insulin sensitivity and fat oxidation [6]. When this coordinated regulation is disrupted, due to insulin resistance, excess nutrient intake, or inflammation, lipid influx to the liver increases. At the same time, oxidation and export are impaired, leading to hepatic steatosis. Over time, oxidative stress, mitochondrial issues, and chronic inflammation contribute to the progression toward NASH and fibrosis [3]. Given the central role of lipid and glucose metabolism in liver homeostasis, noncoding RNAs (ncRNAs) are expected to act at multiple regulatory nodes within these pathways. MicroRNAs (miRNAs) modulate lipid synthesis by targeting transcription factors such as sterol regulatory element-binding protein 1c (SREBP-1c) and carbohydrate-responsive element-binding protein (ChREBP), as well as enzymes like ACC and FAS, thereby influencing de novo lipogenesis [7]. Others, such as miR-33a/b and miR-122, regulate FA oxidation and cholesterol metabolism by targeting CPT1A, PPARα, and ATP-binding cassette subfamily A member 1 (ABCA1) [8]. Long noncoding RNAs (lncRNAs) like H19, MALAT1, and MEG3 influence insulin signaling and mitochondrial β-oxidation through transcriptional or epigenetic mechanisms, while circular RNAs (circRNAs) can serve as “sponges” for lipid-related miRNAs, modulating their downstream effects.

In this context, circRNAs contain multiple binding sites that are complementary to specific miRNAs. By binding or “sequestering” these miRNAs, circRNAs prevent them from interacting with their normal mRNA targets. As a result, the repressive effects of miRNAs on gene expression are reduced, leading to de-repression (upregulation) of the miRNA target genes. In hepatic metabolism and NAFLD, they influence key pathways: circRNA_0046367 sponges miR-34a to enhance PPARα and lipid oxidation, reducing liver fat; circRNA_021412 binds miR-1972, protecting mitochondrial and lipid genes from lipotoxicity; cdr1as sponges miR-7, affecting insulin, lipid storage, and inflammation. As competitive endogenous RNAs (ceRNAs), circRNAs regulate miRNA availability, impacting lipogenesis, β-oxidation, VLDL secretion, and inflammation, all of which are essential for metabolic balance. Dysregulation contributes to NAFLD progression [9].

**Figure 1:**
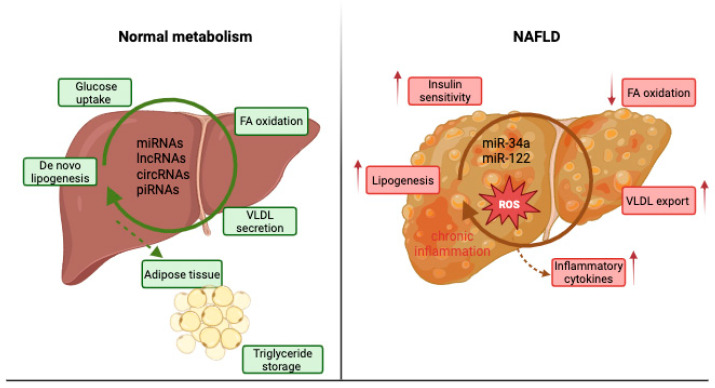
Normal liver metabolism versus NAFLD liver metabolism.

Moreover, ncRNAs are involved in VLDL assembly and secretion, influencing genes such as microsomal triglyceride transfer protein (MTTP) and apolipoprotein B (ApoB), and they participate in the crosstalk between adipose and hepatic tissues by responding to adipokines (leptin, adiponectin) that signal lipid flux and inflammation [6]. Therefore, ncRNAs are essential post-transcriptional regulators that connect metabolic stress to transcriptional reprogramming in hepatocytes, explaining why their altered expression patterns are frequently observed during NAFLD onset and progression [10]. Figure 1 schematically represents the comparative metabolism of the normal liver compared to an NAFLD liver.

Taking into account the many factors are involved in NAFLD pathogenesis, many concepts have been proposed to understand its pathogenesis. The traditional concept underlined that the fundamental basis for the disease initiation is represented by an interplay between genetic and triggering and/or modifying environmental events. In recent years, the important role of epigenetic factors in fatty liver diseases including NAFLD has been increasingly reported [11]. However, the complete picture of NAFLD development and the transition mechanisms from steatosis to NASH are not yet fully understood.

The changes in gene expression determined by mechanisms unrelated to modification of the DNA sequence are shown by epigenetics. Environmental stimuli modulate these mechanisms, and for this reason they are considered reversible phenomena. An imbalance in these epigenic mechanisms can determine different disorders [12]. As a response to different environmental factors, the epigenetic modulation of gene expression can appear in the form of methylated DNA nucleotides or as a modification of histones that determine DNA packing and accessibility. By regulating transcription via altering the activity and stability of mRNAs due to binding of specific ncRNAs such as miRNAs, lncRNAs, circRNAs and piwi-interacting RNAs (piRNAs), epigenetic modulation can occur. Noncoding RNAs are RNAs that result largely from alternative splicing of the more extensive transcripts, which become the precursors for smaller ncRNAs [13]. The ncRNAs are classified into short ncRNAs (they contain under 30 nucleotides) which include miRNAs, circRNAs, piRNAs, and long ncRNAs (which contain over 200 nucleotides) [14]. They are involved in different cellular processes and in a lot of diseases. Current studies demonstrate that ncRNAs are abundantly expressed in the liver, and if their expression is altered different types of liver diseases, including NAFLD, occur. Also, ncRNAs show significant differences in expression related to the severity of NAFLD and histological aspects [15].

Recent advancements in the epigenetics field have proved that epigenetic mechanisms can regulate many aspects of NAFLD pathogenesis. Unlike genetic alterations, epigenetic alterations can be mostly heritable and reversible. So, the use of epigenetic information can serve to identify predictive biomarkers for NAFLD diagnosis and also to find potential therapeutic targets for this disease. In this way, new strategies for early disease diagnosis could be introduced and an optimal individualized patient therapy protocol could be established [16].

Today, the research concerning NAFLD has become urgent and important because the age of NAFLD diagnosis has progressively decreased, and its relationship with the risk of HCC is well known. Because NAFLD ultimately leads to disability and imposes a major socioeconomic burden, timely diagnosis and effective treatment of NAFLD is particularly important [17,18].

In the current review, we present updated information regarding the role of miRNAs, lncRNAs, circRNAs, and piRNAs, with the intention of fostering a good understanding of their regulatory functions in hepatic metabolism and concerning their potential use as biomarkers for early NAFLD/NASH diagnosis and as therapeutic targets.

## 2. MicroRNAs and NAFLD

**Figure 2:**
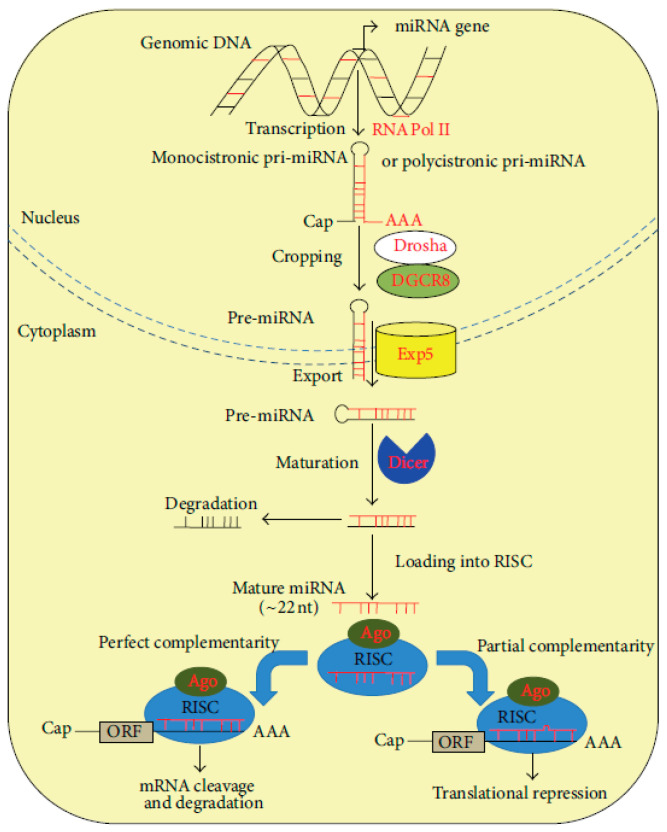
miRNA biogenesis and function [20].

From a structural point of view, miRNAs are small molecules, having a length of approximately 18–22 nucleotides. They are highly conserved short single-stranded ncRNAs whose epigenetic functions are able to transcriptionally regulate gene expression of other RNAs, especially mRNAs. Initially discovered in *Caenorhabditis elegans* in 1993, microRNAs (miRNAs) were later identified across a wide range of organisms, including plants and animals, extending to humans [19]. Current predictions suggest that the human genome encodes approximately 1000 distinct miRNAs, with evidence indicating that these small noncoding RNAs are capable of regulating nearly one-third of all human transcripts [20]. MiRNAs are transcribed from various genomic contexts, including intergenic regions, introns of protein-coding genes and long noncoding RNAs (lncRNAs), and more rarely, from exonic sequences. Notably, some miRNAs are co-transcribed in the same orientation as their adjacent protein-coding genes or host genes, while others are transcribed in the antisense direction [21]. These transcripts are initially synthesized as primary miRNAs (pri-miRNAs), which undergo a two-step maturation process—first within the nucleus and subsequently in the cytoplasm—to generate the functional, mature miRNA molecules (Figure 2) [20].

miRNA genes are transcribed in the nucleus by RNA polymerase II (RNA Pol II) as primary transcripts (pri-miRNAs), which may be either monocistronic or polycistronic. These pri-miRNAs possess canonical 5′ caps (Cap) and 3′ polyadenylated (AAA) tails, characteristic of RNA Pol II transcripts. The initial processing event, known as cropping, is mediated by the Drosha–DGCR8 microprocessor complex, which cleaves the pri-miRNA to release a ≈70 nt precursor miRNA (pre-miRNA) featuring a characteristic hairpin structure. This structure includes a conserved motif that is recognized by the nuclear export receptor Exportin-5 (Exp5), facilitating transport into the cytoplasm. In the cytoplasm, the RNAse III-like nuclease, Dicer, carries out the second processing step (maturation), cleaving the terminal loop of the pre-miRNA to produce a ≈22 nt RNA duplex. This duplex is then unwound, with one strand (the *passenger* strand) typically degraded, while the other (the *guide* strand) is incorporated into the RNA-induced silencing complex (RISC). The core component of RISC is the Argonaute (Ago) protein, which mediates base pairing between the mature miRNA and its target mRNA. Depending on the degree of sequence complementarity to the 3′ untranslated region (3′-UTR) of the target mRNA, mRNA binding leads to either translational repression (in cases of partial complementarity) or mRNA cleavage and degradation (in cases of near-perfect or perfect complementarity). In both scenarios, the ultimate outcome is post-transcriptional gene silencing [21,22].

Concerning the functions of miRNAs, they primarily regulate gene expression by repressing their translation or by promoting miRNA degradation. In this way, they serve as main regulators involved in the control of the expression of thousands of coding and noncoding genes. Many of the conducted studies have shown that more than 60% of human coding genes represent potential targets of miRNAs [23]. More and more studies have demonstrated that dysregulation in miRNAs’ expression is closely related to the molecular processes of different metabolic and liver diseases, including NAFLD. So, by the regulation of several pathogenic processes as altered lipid and glucose metabolism, insulin resistance, and inflammation pathways, miRNAs play important roles in NAFLD development in animal models and humans [24]. Due to the fact that miRNAs can circulate into the microvesicles, exosomes, or apoptotic bodies, they can be bound to RNA-binding proteins and they can be stably detected in biofluids, they have drawn considerable research interest [25].

*miR-10b*

An in vitro miRNA expression profiling study in steatotic human hepatocytes identified miR-10b as a key regulator of lipid accumulation, revealing a novel mechanistic pathway involved in NAFLD pathogenesis. Specifically, miR-10b was found to regulate the nuclear receptor PPAR-α at the post-transcriptional level. PPAR-α plays a central role in fatty acid storage, β-oxidation, and hepatic inflammation, all of which contribute to steatosis. In steatotic hepatocytes, miR-10b was upregulated, and its overexpression led to increased intracellular TG accumulation and lipid content through the direct suppression of PPAR-α [26]. These findings indicate that miR-10b represents a potential therapeutic target for NAFLD. Its dysregulated expression in various cancers has also drawn attention to its role in tumorigenesis, highlighting its therapeutic relevance in oncologic contexts. In particular miR-10b, highly expressed in metastatic HCC tissues and cell lines, constitutes an independent predictor of poor prognosis in patients, promoting migration and metastasis in human hepatocarcinoma cells [27].

*miR-21*

Due to the fact that miR-21 interacts with SREBP1 and 3-hydroxy-3-methylglutaryl-co-enzyme A reductase (HMGCR), it plays an important role in hepatic lipid metabolism by stimulating hepatic lipid accumulation. By targeting low-density lipoprotein (LDL) receptor-related protein 6, miR-21 can inactivate the Wnt/β-catenin signaling pathway, worsening in this way the lipid accumulation and inflammation. miR-21 can target phosphatase and tensin homolog, which are involved in hepatic steatosis prevention, and PPARα expression, which activates lipid oxidation and determines inflammation and fibrosis progression in NAFLD [28]. Studies on diet-induced obese mice showed that miR-21 promotes hepatic steatosis and insulin resistance. These occurred through the regulation of some key transcription factors, such as hepatocyte nuclear factor 4-alpha (HNF4-α), forkhead box protein O1, signal transducer and activator of transcription 3 and insulin-induced gene 2 [29].

In animal models of steatohepatitis and human NASH, dysregulated miR-21 expression was reported. For both NAFLD patients and mouse models, the levels of circulating miR-21 and its expression in the liver were found to be significantly elevated. Also, compared to healthy controls and nonalcoholic fatty liver (NAFL) patients, the circulating miR-21 levels of NASH patients are significantly increased [30]. Other studies demonstrated that inhibiting miR-21 can mitigate steatosis by activating PPARα [31]. Another study carried out on mice with high-fat diet (HFD)-induced steatosis showed that hepatocyte-specific knockout of miR-21 in mice improved steatosis through upregulation of multiple miR-21-targeted pathways involving lipid metabolism. Also, miR-21 abrogation along with obeticholic acid treatment significantly reduced NASH in mice. In HepG2 cells treated with FAs and for diet-induced obese mice miR-21expression was increased. Also, for miR-21 knockout mice fed with a fast food diet, minimal NAFL, inflammation, and apoptosis was found, maybe due to an enhanced expression of PPAR*α* and activation of farnesoid X-activated receptor (FXR) [32].

As a whole, these studies demonstrated that miR-21 has a crucial role in key transitions of NAFLD pathogenesis. These findings recommend miR-21 as a potential serum biomarker for the early detection of patients at risk of developing NASH.

*miR-26a*

Recent studies have uncovered the mechanistic involvement of novel hepatic miRNAs in the progression of NAFLD, underscoring their relevance in human fatty liver disease. One such finding describes a negative feedback loop between miR-26a and PKR-like ER kinase (PERK), a key regulator of the endoplasmic reticulum (ER) stress response. PERK modulates cellular stress by inhibiting its downstream target, eukaryotic initiation factor 2α (eIF2α), thereby attenuating global protein translation to reduce ER load and restore cellular homeostasis [7]. In liver biopsies from individuals with NAFLD, miR-26a expression was significantly downregulated, while markers of endoplasmic reticulum (ER) stress were elevated. Functional studies involving both miR-26a overexpression and silencing in mouse models revealed that, under physiological conditions, ER stress induces miR-26a, which in turn acts to mitigate stress by targeting eIF2α. However, during chronic metabolic stress, as seen in NAFLD, miR-26a expression is suppressed, potentially through a post-transcriptional mechanism, exacerbating ER stress and contributing to metabolic dysregulation in the liver [33].

*miR-29*

Actually, miR-29 is represented by a family of miRNAs which includes as members miR-29a, miR-29b, and miR-29c. The majority of them are expressed in hepatocytes and HSCs [34]. It has been proven that miR-29a is associated with diagnostic relevance in NAFLD, NASH, and liver fibrosis, and also the aggressiveness and prognosis of HCC [24].

It was demonstrated on a mouse model that miR-29a inhibits glycogen synthase kinase 3 beta to repress sirtuin 1 (SIRT1)-mediated mitochondrial biogenesis and improve methionine–choline-deficient diet-induced NASH in mice. Also, miR-29a protects hepatocytes from steatosis by repressing lipoprotein lipase in hepatocytes [35].

Another study proved that miR-29a disrupts DNA methyltransferase 3β (DNMT3β) and improves diet-induced NASH in mice. Also, miR-29a suppresses the cluster of differentiation 36 (CD36) and plays a regulatory role in NAFLD by improving HFD-induced steatohepatitis and liver fibrosis [24]. The conclusion of the study was that miR-29 family members are downregulated in mouse models of liver fibrosis and in human fibrotic livers. In humans, drug-induced NAFLD can be predicted by identifying circulating miR-29 as a potential biomarker. In NAFLD patients, serum miR-29a levels are lower than those of controls [36].

*miR-33*

In humans, miR-33 is a family formed of miR-33a and miR-33b. Their targets are represented by SREBP1/SREBP2 and ABCA1. They are co-transcribed with SREBP1 and SREBP2. miR-33a/b is involved in fatty liver disease and participates in lipid transport and metabolism by targeting some genes that have roles in insulin signaling pathways and in cholesterol homeostasis [37].

In mice, there is only one miR-33 isoform. This is an ortholog form of human miR-33a [38]. A study on mice demonstrated that miR-33 regulates liver lipogenesis signaling and that miR-33 can be used as a potential circulating biomarker for NAFLD. The treatment with anti-miR-33 therapeutic agents in mouse models of atherosclerosis can reduce plaque burden and offers hope for a therapeutic perspective in treating cardiovascular diseases. Long-term therapeutic silencing of miR-33 in mice causes adverse effects such as hypertriglyceridemia and hepatic steatosis. Hepatic steatosis and worsening of obesity was recorded for miR-33 knockout mice exposed to HFD via targeting SREBP1. The researchers concluded that the genetic loss of miR-33 results in an increase in food intake and determines obesity and insulin resistance [39].

In the liver tissues of the patients diagnosed with NAFLD, the expression levels of miR-33 are increased. For NASH patients with morbid obesity, the expression of hepatic miR-33a is also increased. A published clinical trial demonstrated that an increased expression of miR-33a in the liver is related to the presence of steatohepatitis in morbidly obese humans and in metabolic dysfunction [37,40]. In patients with NAFLD after liver transplantation, circulating miR-33a is associated with steatosis and inflammation and can be used as a predictor for these pathological conditions [41]. To understand the role of miR-33a/b in NAFLD, more studies are needed in order to provide new insights into the physiopathology of various forms of the disease.

*miR-99 a/b*

The miR-99a/b family is a family of tumor suppressor miRNAs. miR-99a ranks as the sixth most abundant microRNA in the normal human liver miRNome, yet it is substantially downregulated in hepatocellular carcinoma (HCC). Its tumor-suppressive function is primarily attributed to its ability to induce cell cycle arrest, thereby inhibiting tumor proliferation. Given these properties, miR-99a has emerged as a promising prognostic biomarker for HCC [42]. The downregulation of miR-99a/b in the adipose tissue of obese individuals and NAFLD patients has been consistently reported. Specifically, miR-99a levels were inversely correlated with FFA and IL-6 serum levels. Moreover, miR-99b, secreted by visceral adipose tissue in NAFLD patients, was found to be significantly associated with pericellular fibrosis in patients with NASH. These findings suggest that miRNA expression profiles from visceral adipose tissue may serve as potential biomarkers to distinguish simple steatosis from NASH [43].

*miR-122*

miR-122 represents about 70% of the total miRNAs produced by the liver, and it is the most studied. miR-122 has an important role in liver function and in the epigenetic modulation of several genes linked to chronic pathology of the liver [44]. miR-122 is involved in the regulation of lipid metabolism. Experiments carried out on mice showed that the inhibition of miR-122 enhances FA oxidation, determines a decrease in the rate of hepatic FAs and cholesterol biosynthesis, a reduction of cholesterolemia, and acts as a protection for the HFD-fed mice across hepatic steatosis. It was reported that miR-122 targets specific genes involved in cholesterol biosynthesis, such as HMGCR, microsomal TG transfer protein, FA synthase, 3-hydroxy-3-methylglutaryl-coenzyme A (CoA) synthase 1, and acetyl-CoA carboxylase. This suggested the role of miR-122 in NAFLD pathogenesis [45]. Specific to NAFLD is the excessive accumulation of TG in the hepatocytes’ cytoplasm. Genetic deletion of miR-122 locus in mice causes TG accumulation in the hepatocytes and consecutively hepatic steatosis that progresses to NASH, fibrosis, and HCC. In contrast, restoration of miR-122a expression reduces disease symptoms and tumorigenesis. Similar to what was obtained in animal studies, reduced expression of miR-122 is also registered in the hepatic tissues of NASH patients, compared to patients with simple steatosis or healthy controls [44]. Changes in miRNA expression profiles were found at various stages of NAFLD, from simple fatty liver, NASH, liver fibrosis, to HCC. In this context, a study conducted with NAFLD patients reported that the hepatic miR-122 levels were lower in patients with mild steatosis compared to those with severe steatosis and that serum and hepatic miR-122 levels were significantly higher in patients with mild fibrosis than in those with severe fibrosis. Compared to controls, elevated serum levels of miR-122 were found in NAFLD patients, and these levels are positively correlated with the severity of the disease [45]. Other studies demonstrated that circulating levels of miR-122 are positively correlated with fatty liver disease, obesity, T2DM, and atherosclerosis. Moreover, NASH patients have an increased level of miR-122 in the serum and a decreased hepatic expression of this RNA [46,47]. miR-122 expression differs between hepatocytes and blood. The mechanisms underlying such an inverse correlation are complex and need more investigation. For now, it is known that elevated levels of circulating miR-122 may be attributed to its secretion via liver exosomes. Taking into account that the dynamic of miRNA’s expression, secretion, and transport is complex, other tissues, like the adipose tissue, can contribute to the pool of miR-122 levels [45].

*miR-128-2*

miR-128-2 is recognized as a proapoptotic miRNA that inhibits cancer cell invasion and functions as an endogenous negative regulator of SIRT1, thereby influencing the p53 signaling network. More recently, miR-128-2 has been implicated in the regulation of cholesterol homeostasis, acting to suppress cholesterol efflux by targeting the key transporters ABCA1, ABCG1, and retinoid X receptor alpha (RXRα) in hepatic cell lines and in liver tissues from high-fat diet (HFD)-fed mice, where its expression was found to be downregulated. This downregulation was associated with increased intracellular cholesterol accumulation, partially mediated by the upregulation of SREBP-2, suggesting a potential feed-forward loop enhancing miR-128-2 expression. The reduction of miR-128-2 in obese mice may contribute to apoptosis resistance and promote tumorigenesis. Therefore, by promoting hypercholesterolemia, a common feature in obesity, miR-128-2 may represent a critical molecular link between obesity and cancer. However, miR-128-2’s role in the context of NAFLD requires further investigation [48].

*miR-144*

miR-144 has been recently shown to be upregulated specifically in liver macrophages and hepatocytes from obese and insulin-resistant mice and humans. This upregulation impairs the antioxidant response to hepatic lipid accumulation by targeting nuclear factor erythroid 2-related factor 2 (NRF2), a key regulator of cellular redox homeostasis. miR-144 suppresses NRF2 function through two mechanisms: directly by reducing NRF2 protein expression, and indirectly by downregulating immunoresponsive gene 1 (IRG1). The latter leads to alterations in tricarboxylic acid (TCA) cycle metabolites, ultimately resulting in further inhibition of NRF2 activity [49]. Interestingly, the transcription factor GATA-binding protein 4 (GATA4) was identified as a key driver of miR-144 upregulation in the livers of obese, insulin-resistant individuals compared to lean controls. This increased transcriptional activity appears to be mediated, at least in part, by activation of the extracellular signal-regulated kinase (ERK) signaling pathway [50].

*miR-155*

miR-155 is a multifunctional miRNA which regulates important processes like lipid metabolism, immunity, inflammation, and cancer. For miR-155-deficient mice fed with a HFD, an increased hepatic steatosis takes place when compared to controls. For the same mice, liver-specific overexpression of miR-155 determines a reduction of serum and hepatic levels of TG, total cholesterol (TC), and high-density lipoprotein (HDL), and consecutively mitigates NAFLD symptomatology [51]. This means that miR-155 plays a protective role in NAFLD and its pathological conditions. For miR-155 knockout mice fed with a methionine-choline-deficient diet, a reduction in the expression of genes involved in FA metabolism and fibrosis took place, with a decrease in steatosis. No inflammation or liver injuries were present [52]. For miR-155 knockout mice fed with a fat diet rich in cholesterol and sucrose, decreased steatosis, fibrosis attenuation, and less liver injury was found compared to control mice [53]. The ambiguous roles of miR-155 suggest that it may exert pleiotropic functions influenced by the underlying etiology and disease state. Variability in reported outcomes may stem from the differential release of miR-155-containing exosomes or microvesicles into surrounding tissues. In murine models, adipose tissue-derived miR-155, upregulated by an HFD, has been shown to contribute to hepatic insulin resistance. Notably, miR-155 has emerged as one of the most relevant microRNAs implicated in liver diseases including NAFLD [54]. For NAFLD patients, it was demonstrated that miR-155 level is decreased in liver tissue and peripheral blood, compared with healthy controls. The decreased miR-155 activity in NAFLD patients may be due to the adipogenic transcription factors CCAAT/enhancer binding protein (C/EBP)-α, C/EBP-β, PPAR-γ, and liver X receptor (LXRα) [52]. Further studies are needed to clarify the contradictory results and to determine the role of miR-155 in intracellular lipid accumulation and NAFLD development and progression.

*Let-7 family*

In the context of NAFLD, the expression of different let-7 family members appears to be differentially regulated. Notably, let-7b is upregulated in steatohepatitis compared to steatosis, while let-7d shows the opposite trend, being downregulated in steatohepatitis. These findings suggest that individual let-7 miRNAs may undergo distinct regulatory changes during NAFLD progression and could potentially serve as predictive biomarkers [55]. The let-7 miRNA family is well known for its critical roles in liver fibrosis and tumorigenesis, primarily by protecting human hepatocytes against oxidative stress and acting as potential suppressors of cell proliferation. For instance, recent studies have shown that let-7b and let-7c can indirectly enhance the expression of heme oxygenase 1 (HMOX1), a key cytoprotective enzyme, thereby reducing oxidative damage in hepatocytes. These findings suggest that overexpression of specific let-7 members could offer a promising therapeutic strategy to safeguard hepatocytes from oxidative injury, a key event in the progression and exacerbation of liver diseases such as fibrosis and HCC [56]. In contrast, let-7e, another member of the let-7 family, has been associated with the progression of liver fibrosis in mouse models. Regarding the role of the let-7 family in liver cancer, studies in 32 HCC patients revealed that let-7c expression was significantly lower in liver tumor tissues compared to adjacent non-tumorous tissues. Moreover, this downregulation of let-7c was positively correlated with poor histological differentiation in HCC. Similarly, let-7g expression was reduced in human HCC cell lines. Functional studies involving transfection of hepatocarcinoma cells indicated that let-7g may act as a tumor suppressor, inhibiting HCC cell proliferation through downregulation of c-Myc and upregulation of the tumor suppressor gene p16 (INK4A). Conversely, other family members, such as let-7a and let-7b, were found to be upregulated in hepatic cancer stem cells (CSCs), suggesting their potential as molecular targets for HCC eradication [57].

*miR-181 a/b*

In a recent study using mouse models of NAFLD, plasma levels of miR-181a were found to be correlated with both susceptibility to NAFLD and the severity of liver injury associated with the disease. More recently, miR-181b has emerged as a potential diagnostic serum biomarker for liver cirrhosis in humans. Specifically, miR-181b was shown to be induced by transforming growth factor-β1 (TGF-β1) and to promote HSCs proliferation by directly targeting the cyclin-dependent kinase inhibitor 1B (p27). Elevated serum levels of miR-181b were observed in cirrhotic patients compared to healthy controls. Additionally, miR-181 was found to be upregulated in CD133^+^, epithelial cell adhesion molecule (EpCAM)^+^, and AFP^+^ tumor-initiating stem cells (TISCs) based on miRNA expression profiling in HCC. This upregulation appears to be driven by activation of the wingless (Wnt)/β-catenin signaling pathway, a key regulator of CSCs origin and malignancy in benign adenomas. Furthermore, this pathway also influences miR-181 expression through activation of the β-catenin target oncogene c-Myc [58,59]. Furthermore, a recent study conducted on hepatocellular CSCs using global microarray-based miRNA expression profiling followed by real-time PCR (RT-PCR) validation revealed that conserved miR-181 and let-7 family members were upregulated in these cells. Their expression was shown to be regulated by IL-6 and the basic helix-loop-helix transcription factor Twist. The same study demonstrated that these miRNA families play a crucial role in tumor progression, and that their inhibition enhanced the responsiveness to chemotherapy, highlighting their potential as therapeutic targets in HCC [57].

*miR-192*

miR-192 promotes fibrogenesis and is involved in fibrosis development and TGF*β*/SMAD signaling activation. For NASH patients, liver miR-192 is downregulated and serum levels of miR-192 are increased by ≈4-fold compared to controls. In human and animal models, during pathophysiological conditions miR-192 is released from hepatocytes, possibly due to membrane damage. This suggest that miR-192 has a potential to be used as a NASH biomarker [60].

*miR-34a*

miR-34a is a member of the miR-34 family, which also includes miR-34b and miR-34c. For patients with NAFLD and NASH, miR-34a expression levels are increased in the serum and also in the liver. miR-34a expression levels are positively correlated with TG and TC levels. Many transcription factors, such as SIRT1, HNF4-α and p53, which are involved in lipid metabolism, fatty acid β-oxidation, and cholesterol synthesis, are regulated by miR-34 [61]. It was demonstrated in a mouse NASH model that the miR-34a/SIRT1/AMP-activated protein kinase (AMPK) pathway is involved in mitochondrial dysfunction. For mice fed an HFD and for NASH patients, miR-34a inhibits the secretion of hepatic LDL by promoting steatosis through interaction with HNF4-α. In NAFLD patients, miR-34a regulates steatosis by targeting PPARα expression [62]. For mice fed an HFD and for NASH and NAFLD patients, higher circulating levels of miR-34a have been found. Among patients with NAFLD, an association of miR-34a and miR-122 with dyslipidemia was reported. Both miRNAs could be useful as biomarkers in patients with obesity and NAFLD [63]. In a meta-analysis study, miR-34a, miR-122, and miR-192 were identified as potential diagnostic markers to separate NAFL from NASH. In this case, miR-34 proved to have the best diagnostic value [24].

*miR-16*

miR-16 is a 21-nucleotide miRNA that has been found to be overexpressed in both rat and human models of NAFLD/NASH compared to healthy controls, with its levels correlating with the degree of liver inflammation. Along with miR-34a and miR-122, circulating miR-16 has emerged as a potential non-invasive biomarker for assessing disease stage in NAFLD, as its expression was significantly elevated in NAFLD patients relative to healthy individuals and was associated with the disease’s severity. Moreover, a pronounced upregulation of miR-16 has been observed during the progression from NASH to HCC, suggesting a role in disease advancement [64]. However, other studies have reported downregulation of miR-16 during HSCs activation. This was further supported by in vitro experiments, where miR-16 overexpression significantly inhibited HSC proliferation, induced apoptosis, and ultimately reduced hepatic fibrosis. Current literature highlights the key role of miR-16 in NAFLD pathogenesis, suggesting its potential as a prognostic marker for the disease. Although findings from HCC patients indicate that miR-16 may lack specificity for NAFLD, its use in combination with other more specific miRNAs could enhance diagnostic and prognostic accuracy in clinical settings [20].

*miR-199 a/b-3p*

Recent microarray and bioinformatics analyses conducted to identify the specific miRNA profile involved in the transition from steatosis to steatohepatitis in a rat model fed a high-fat diet revealed that miR-199a is dysregulated during the progression of liver inflammation from simple steatosis to steatohepatitis [55]. miR-199a levels have been shown to be closely correlated with the severity of liver fibrosis in both humans and mice, with significantly higher expression observed in advanced fibrosis compared to healthy controls. Among tumor-suppressive microRNAs, miR-199a/b-3p ranks as the third most abundantly expressed miRNA in normal human livers and has been found to be markedly downregulated in HCC. Notably, low miR-199a/b-3p expression has been identified as an independent predictor of reduced tumor-free survival in HCC patients, further supporting its critical role in suppressing HCC progression [65].

*miR-200*

The miR-200 family includes miR-200a, miR-200b, miR-200c, miR-141, and miR-429. Among these, miR-200a and miR-200b were found to be upregulated in rat models of hypercaloric diet-induced NAFLD compared to controls. This dysregulation was associated with increased body weight and alterations in both histological and metabolic parameters characteristic of NAFLD. Notably, these miRNAs were shown to target genes involved in apoptosis, lipid, and carbohydrate metabolism pathways, with corresponding proteins exhibiting aberrant expression in animals fed with an hypercaloric diet. The link between miR-200b expression and the pathophysiological features of NASH was further supported in a mouse model mimicking human NASH. In mice fed with a lipogenic, methyl-deficient diet, a distinct expression profile of miRNAs and their target genes was observed relative to controls. The progression of NASH in this model was accompanied by upregulation of miR-200b, miR-34a, and miR-155 along with downregulation of miR-29c, suggesting that the severity of and susceptibility to diet-induced NASH may be determined by specific alterations in miRNAs’ expression. Consistent findings were also reported in diverse strains of mice fed with a choline- and folate-deficient diet [58]. In this study, circulating levels of several miRNAs, including miR-200b, showed a significant correlation with the severity of NAFLD-associated liver injury, highlighting their potential as highly sensitive plasma biomarkers for the non-invasive monitoring of NAFLD progression and liver damage extent. In both mouse models and human liver fibrosis specimens, the expression of 11 microRNAs was found to be associated with disease progression. Notably, the upregulation of four miRNAs (miR-199a/b-3p, miR-200a, and miR-200b) showed a significant positive correlation with the degree of fibrosis. Moreover, overexpression of these miRNAs in HSCs led to a marked increase in the expression of fibrosis-related genes, emphasizing their functional role in fibrogenesis. Collectively, these findings highlight the therapeutic and diagnostic potential of the miR-200 family in hepatic fibrosis. Additionally, the miR-200 family is known for its involvement in HCC metastasis pathways. In particular, studies have shown that in various HCC cell lines, miR-200a and miR-200b regulate cell migration by targeting the expression of E-cadherin, a key cell–cell adhesion molecule, thereby influencing metastatic potential [66]. It was concluded that the miR-200 family may represent a promising therapeutic target against hepatocarcinoma metastasis. Both in vitro and in vivo studies have demonstrated that epigenetic activation of the miR-200 pathway by the long noncoding RNA H19 can reverse the epithelial–mesenchymal transition response, thereby suppressing the rate of tumor metastasis, even in aggressive forms of HCC [67].

*miR-221*

Interestingly, miR-221 is among the miRNAs found to be upregulated in the livers of ob/ob mice, indicating a potential role in the development of NAFLD. Its expression pattern, observed in both mouse and human liver samples, was positively associated with the degree of fibrosis, increasing as fibrosis progressed. This suggests its involvement in fibrosis regulation, in contrast to miR-29 family members, which were notably downregulated in fibrotic and advanced cirrhotic patients. Consistently, both in vitro and in vivo studies have identified miR-221 as a potential biomarker for HSCs activation and for the progression of liver fibrosis [68]. miR-221 has been shown to be dysregulated in the early stages of NASH-induced HCC in mice and is actively involved in hepatocarcinogenesis. These findings underscore its key role in NAFLD progression and highlight its potential prognostic value. Classified as an oncomiR, miR-221 is among the most upregulated miRNAs in HCC, as demonstrated in both transgenic mouse models [69] and human studies, where approximately 70–80% of HCC cases exhibited its overexpression. Moreover, elevated serum and tissue levels of miR-221 have been associated with cirrhosis, advanced tumor stage and size, metastasis, and reduced survival rates in HCC patients compared to healthy individuals. These associations reinforce the diagnostic and prognostic significance of miR-221 for HCC and support its potential as a therapeutic target in liver cancer [70].

*miR-370*

miR-370 is a good post-transcriptional regulator for lipid metabolism. Carnitine palmitoyltransferase 1A (CPT1A), an enzyme involved in FA oxidation, is directly targeted by miR-370. miR-370 may have a role in the accumulation of TGs in the liver through the modulation of miR-122 expression. In HepG2 cells, overexpression of miR-370 activates FAS and acetyl-CoA carboxylase 1 (ACC1) via modulation expression of SREBP-1c. These enzymes are also involved in lipogenesis [71].

*miR-378*

miR-378 has been implicated in the pathogenesis of both early and advanced stages of NAFLD. Its expression is upregulated in the livers of diet-induced obese (DIO) mice, as well as in human NAFLD/NASH samples. Notably, LXRα has been shown to uncouple miR-378 expression from that of its host gene, PPARγ coactivator 1-beta (PGC1β), a positive key regulator of mitochondrial function and fatty acid oxidation, by activating miR-378 transcription while simultaneously suppressing PGC1β transcription [72]. In addition, earlier studies on mice demonstrated that miR-378 directly targets the transcription factor and mitochondrial regulator nuclear respiratory factor 1 (NRF1), thereby inhibiting FA oxidation [73]. Consequently, elevated hepatic miR-378 levels may support the activity of its own transcriptional activator, LXRα, in driving the development of hepatic steatosis by suppressing key regulators and effectors of lipid metabolism in the liver. Another direct target of hepatic miR-378 is PRKAG2, the gene encoding the γ2 subunit of AMPK. Furthermore, miR-378 has been implicated in the pathogenesis of NASH, where it contributes to hepatic inflammation, necrosis, and apoptosis. This occurs through the activation of nuclear factor kappa B (NF-κB)/tumor necrosis factor (TNF) inflammatory signaling, mediated by the repression of AMPK and its downstream effector, SIRT1, a known inhibitor of the NF-κB subunit p65 [74].

*miR-375*

Due to the fact that miR-375 is highly expressed in pancreatic islets, it is considered to be an important regulator of insulin secretion and implicitly of glucose homeostasis [75]. miR-375 is involved in the pathogenesis of NAFLD. In palmitate-induced HepG2 cells, miR-375 inhibition suppresses the production of TNF and IL-6 and increases adiponectin expression. In these ways, lipid accumulation in suppressed. In NAFLD patients, miR-122, miR-192, and miR-375 are significantly upregulated compared to controls. These data suggest that miR-375 could be a promising target for NAFLD progression and prevention [76].

In conclusion, miRNAs play a central regulatory role in the pathogenesis, progression, and potential treatment of NAFLD. They modulate gene expression at the post-transcriptional level, either by degrading target mRNAs or inhibiting their translation. In the case of the patients diagnosed with NAFLD, different miRNA signatures in liver tissue and peripheral blood were found. In response to high fat deposition, NAFLD patients’ hepatocytes become insulin-resistant and HCs are responsible for initiating protective or pathogenic signals. miRNA’s importance related to NAFLD pathogenesis include the following aspects: regulation of lipid metabolism, which represents the key in steatosis, involvement in inflammation and transition to NASH, oxidative stress and apoptosis, fibrosis, and hepatic stellate cell activation. miRNAs represent potential non-invasive biomarkers to replace or complement liver biopsy. In this way, they could become biomarkers for non-invasive diagnosis. Also, miRNAs could be used as therapeutic targets with the aim of reducing steatosis and liver injury and restoring normal lipid metabolism.

## 3. Long Noncoding ARNs and NAFLD

lncRNAs are a group of molecules longer than 200 nucleotides that are not generally involved in protein-coding and are therefore often referred to by scholars as ‘noisy sequences’ [77]. lncRNAs are transcribed from the same genes as miRNAs and, following splicing, they also possess a 5′ cap and a 3′ poly(A) tail. Through alternative splicing, they can generate distinct transcripts from the same gene. However, unlike miRNAs, lncRNAs lack protein-coding capacity. They also differ in terms of cellular localization and abundance, with various lncRNAs found at different levels both within and outside the cell [78].

**Figure 3:**
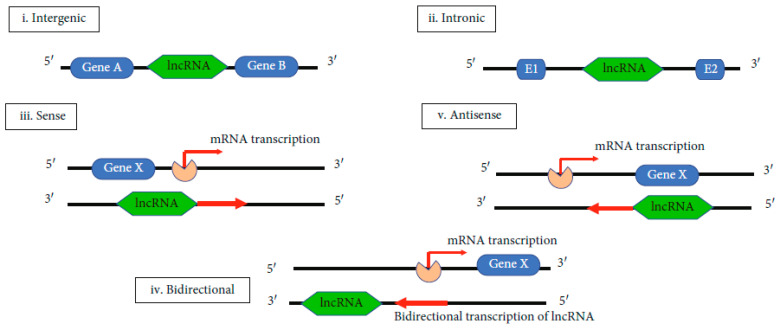
Biogenesis of lncRNAs [71].

The biogenesis of lncRNAs is not entirely elucidated. Its elucidation is important for understanding its functional significance and to define the difference between lncRNAs and other types of RNAs. LncRNA is cell type-specific and it is under the control of cell type-stimuli. It is also stage-specific, being under the control of stage-specific stimuli. The molecular mechanisms underlying lncRNA’s biogenesis are still under study. It is known that lncRNA can be transcribed by the RNA polymerase II from exonic, intergenic, or the distal protein-coding regions of the genome to produce the premature lncRNA. Premature lncRNA is polyadenylated in position 3′ and capped with methyl-guanosine in position 5′. It is possible that histone methylation plays an important role in lncRNA biogenesis. The premature lncRNAs can suffer alternative splicing to form different proteins [79,80]. Five types of lncRNAs can be obtained, depending on the region of transcription. These are: i—intergenic, ii—intronic, iii—sense, iv—bidirectional and v—antisense (Figure 3) [71].

Small RNA deep sequencing data indicate that lncRNA could also encode small functional RNA [81]. Mature lncRNAs can be located in the cytoplasm or/and in the nucleus. Even if the cytoplasmic lncRNAs are not translated, lncRNAs interacts with ribosomes and produces small peptides, which have been identified [82]. LncRNAs can have both trans- and cis-regulatory activity. As trans-regulators, lncRNAs have the possibility to control gene expression located at a distance from their transcription site by influencing the nuclear structure, by altering the chromatin state, or by regulating protein function. As cis-regulators, lncRNAs affect neighboring genes on the same allele from which they are transcribed [83].

Researchers have demonstrated that lncRNAs exert their influence through several key mechanisms: (a) guiding target localization; (b) serving as molecular scaffolds to mediate protein-RNA interactions; (c) functioning as miRNA sponges; (d) acting as molecular decoys by binding directly to proteins, thereby inhibiting downstream gene expression; (e) modulating chromatin remodeling and histone modifications by encoding transcripts from upstream promoters of target genes, and (f) serving as precursors for small RNAs. Additionally, studies have confirmed that lncRNAs participate in a broad range of biological processes, including epigenetic regulation, the regulation of protein complexes, chromosome recruitment, and inactivation, and the control of cell growth and apoptosis [84].

The liver plays the most important role in maintaining metabolic homeostasis, being the main place where biosynthesis, storage, metabolism, and redistribution of carbohydrates, lipids, and proteins take place [85]. Numerous studies have shown that many lncRNAs represent key regulators of glucose and lipid metabolism. The balance between glucose biosynthesis and/or its storage in the liver and the uptake of glucose in the peripheral tissues is responsible for glucose homeostasis maintenance. Concerning lipid metabolism, the balance between catabolic processes such as fatty acid β-oxidation, lipolysis, lipogenesis, and thermogenesis are responsible for lipid accumulation in the liver. Disturbances in the regulation of these processes determine dyslipidemia, adiposity, and metabolic disorders [86].

Numerous dysregulated lncRNAs have been identified in NAFLD, where they play critical roles in processes such as lipid accumulation, the progression to NASH, NASH-associated fibrosis, and HCC. These effects are mediated through various mechanisms, including functioning as ceRNAs, interacting with RNA-binding proteins, and regulating their post-translational modifications by phosphorylation, acetylation and ubiquitination.

To date, numerous abnormally expressed lncRNAs have been identified in NAFLD. Studies have revealed that 1735 lncRNAs and 1485 mRNAs are differentially expressed in NAFLD samples compared to healthy liver tissues. These molecules are extensively involved in hepatic lipid metabolism, the development of NASH, NASH-associated fibrosis, and NAFLD-related HCC [87].

*lncRNA H19 (H19)*

H19 is one of the first discovered lncRNAs, and it is considered to be a transcription product of the H19 gene. The predominant role of H19 is to affect miRNA’s stability in different pathological and physiological conditions. In the last few years, due to its aberrant expression and its extensive involvement in several hepatic metabolic processes, H19 has gained increased attention in the research of liver diseases [88]. Studies showed that the overexpression of H19 results in hepatic metabolic reprogramming and worsens diet-induced fatty liver. It has also been demonstrated that expression of H19 activates the mammalian target of rapamycin complex 1 signaling and the lipogenic transcription factor MLX interacting protein-like pathways, which are involved in hepatic steatosis production. The knockdown of H19 in NAFLD animal models caused steatosis inhibition and a reducing of hepatic lipogenesis by acting at the PPARγ level [89]. More studies are needed to elucidate the role of H19 in NAFLD pathogenesis [90].

*lnc18q22.2*

lnc18q22.2 is a liver-specific lncRNA, involved in growth, mRNA translation, viability, oxidation–reduction processes, and cell death of liver cells. Besides the fact that lnc18q22.2 is expressed in the liver, RT-PCR analysis proved that lnc18q22.2 is expressed also in liver cell lines, like HepG2, Hep3B, Huh7, and primary human hepatocytes compared with HeLa and HEK293T cells. The knockdown of lnc18q22.2 in NAFLD produces a decrease in cell viability or lethal phenotype in hepatocytes cell lines. Data indicates that an elevated level of lnc18q22.2 expression negatively regulates the genes involved in oxidation–reduction processes. The knockdown of lnc18q22.2 downregulates anti-apoptotic genes, including BCL2 family proteins. These effects leave behind a necrosis-like phenotype in the liver as a result of lnc18q22.2 knockdown. For patients with steatohepatitis on whom a biopsy was done, an increased level for lnc18q22.2 expression was found. Taking into account this data, lnc18q22.2 may become a new therapeutic target for NASH treatment [91].

*lncRNA HCV regulated 1 (lncHR1)*

lncHR1 is a human-specific lncRNA involved in lipid metabolism. In an HFD mouse model, overexpression of lncHR1 lowered oleic acid-induced hepatic cell TGs and lipid droplets’ accumulation and inhibited FAS by inhibiting *SREBP1c* gene expression. These results are relevant for NAFLD patients because dyslipidemia in their case has an atherogenic nature and is characterized by hypertriglyceridemia. Moreover, hypertriglyceridemia is associated with cardiovascular disease and metabolic syndrome [92].

*Runt-related transcription factor 1 (RUNX1)*

Inflammation and pathological angiogenesis derived from oxidative stress are involved in the progression of NAFLD to NASH, NASH to cirrhosis, and finally to HCC. RUNX1 was studied for its role in NAFLD, because it has been proposed as a regulator of angiogenesis (via vascular endothelial growth factor (VEGF)), hematopoiesis, and inflammation (tool-like receptor 4 (TLR4)-mediated inflammation) [93,94]. Immunohistochemical and qRT-PCR analyses indicated that RUNX1 and its target genes (CCL2 and PIK3CA) are positively correlated with the degree of steatosis, inflammation, and fibrosis. Knockdown of RUNX1 in human umbilical vein endothelial cells (HUVEC) affected adhesion molecules such as VEGF, vascular cell adhesion molecule-1 (VCAM1), platelet-endothelial cell adhesion molecule-1 (PECAM1), CCL2, and mRNA expression of chemotactic and angiogenic factors. RUNX1 increases the angiogenic activity of the HUVECs cell line. As a conclusion, the RUNX1 mechanism of action in liver diseases is based on the upregulation of its downstream genes, including VEGFs, adhesion molecules, and chemokines. RUNX1 expression is correlated with the severity of NAFLD. It has been suggested that RUNX1 targeting could overcome the exacerbation of FA-related liver diseases [95].

*Ultra-conserved element (UC372)*

UC372 may play a role in NAFLD pathogenesis because it is an lncRNA associated with an impaired lipid metabolism homeostasis. UC372 can produce hepatic steatosis by binding to pri-miR-195/pri-miR-4668, thus preventing miR-195/miR-4668 from regulating the expression of target genes associated with lipogenesis and lipid uptake, including FAS, ACC, stearoyl-CoA desaturase 1, and lipid uptake-related genes like CD36. In the case of HFD-fed mice, in a murine model of T2DM, and for NAFLD patients, UC372 is upregulated. This indicates the role of UC372 in liver steatosis and fatty liver. The obtained results suggested that UC372 could be used as a promising target for hepatic steatosis therapy [96].

*lncRNA activated in renal cell carcinoma (RCC) with sunitinib resistance (lncARSR)*

lncARSR is a lncRNA formed by 591 nucleotides and it has been studied mostly in cancer, especially renal and hepatocellular carcinoma [97]. Concerning liver diseases, in vitro studies have demonstrated that lncARSR overexpression induces the expression of lipogenic genes like FAS, stearoyl-CoA desaturase 1 (SCD1), and SREBP-1c. Through the Akt/SREBP-1c pathway, lncARSR controls hepatic lipogenesis, which provides new data about the metabolic role of lncARSR. In in vivo studies carried out on mice, lncARSR levels were elevated in the liver of methionine-choline deficient (MCD) mice compared to chow diet-fed mice. For NAFLD patients, lncARSR levels are elevated both in the serum and in the liver [98]. The expression of lncARSR is increased in patients with hypercholesterolemia and for mice fed with a high-cholesterol diet. The knockdown of lncARSR in a Hep-G2 cell line and in a murine model demonstrated that cholesterol metabolism is modulated by lncARSR. lncARSR also modulates HCC resistance to doxorubicin via phosphatase and the tensin homolog—phosphoinositide 3-kinases (PTEN-PI3K)/Akt pathway [97]. lncARSR specifically binds and blocks yes-associated protein 1 (YAP1) phosphorylation. In this way, YAP1 is imported into the nucleus. The YAP1 phosphorylation blockade determines the activation of YAP1. It has been concluded that YAP signaling pathways promote the progression and development of NAFLD [99].

*Fatty liver-related lncRNA 2 (FLRL2)*

FLRL2 is a lncRNA located in the intronic region of the aryl hydrocarbon receptor nuclear translocator-like 1 protein (ARNTL) gene. ARNTL is considered as a cis target of FLRL2. FLRL2 has been identified as an important compound for NAFLD pathogenesis. In a NAFLD mouse model, FLRL2 is downregulated, suggesting its involvement in NAFLD pathogenesis. For HFD mice, the overexpression of FLRL2 improves NAFLD by activation of the ARNTL-SIRTA pathway, and in this way inhibits lipogenesis and implicitly reduces hepatic steatosis. The obtained results suggest that FLRL2 can become a candidate for NAFLD treatment [100].

*lncRNA NONMMUT010685 and NONMMUT050689*

ATP citrate lyase (ACLY), plays an important role in FA biosynthesis by converting citrate to acetyl-CoA. In animals, it links this cycle to carbohydrate metabolism. ACLY activity has been correlated with dyslipidemia, reduced tolerance to glucose and metabolic disorders, including hepatic steatosis. In NAFLD, ACLY enzyme is significantly increased [101]. In ACLY knockdown model mice and in leptin-deficient mice, the TG and VLDL levels were found to be decreased [102]. NONMMUT010685 and NONMMUT050689 are lncRNAs increased in NAFLD. These two lncRNAs were proposed as regulators of X-box binding protein (XBP1) and receptor-interacting protein kinase 1 (RIPK1) [101]. XBP1 is considered to be a key regulator of unfolded proteins and has an important function in human dyslipidemias, being essential in the maintenance and development of secretory cells, which are correlated with janus kinase (JNK) activation. Due to the fact that the proteins are not sufficiently catabolized after XBP1 activation, NASH patients are at risk of developing hepatic cirrhosis. RIPK1 is associated with inflammation and cell death pathways. By its kinase activity, it initiates RIPK3-mediated necroptosis. The role of RIPK1 in NASH development it is that it limits the progression of liver fibrosis [103]. It has been demonstrated that in NASH, XBP1 and RIPK1 are downregulated, indicating their involvement in NASH pathogenesis. As a conclusion, the upregulation of NONMMUT010685 and NONMMUT050689 in NAFLD downregulates XBP1 and RIPK1, increases ACYL enzyme, and causes NASH development [101].

*Alu-mediated transcriptional regulator (APTR)*

APTR is an lncRNA involved in the regulation of cell cycle progression and proliferation. APTR has been demonstrated to be highly expressed in two animal models of liver fibrosis, namely in liver tissue treated with CCl4 and in bile duct ligation mice, and also in patients with liver fibrosis. The knockdown of APTR suppressed the activation of HSCs in vitro and diminished the in vivo accumulation of type 1 collagen chain (COL1A1). In the serum of patients diagnosed with liver cirrhosis, APTR levels are increased. This suggests that APTR can become a biomarker for liver cirrhosis. There are also some data that indicate the role of APTR in hepatofibrogenesis. To confirm them, new studies to analyze serum APTR in large cohorts of patients are needed [104].

*lncRNA-COX2*

From the structural point of view, cyclooxygenase 2 (COX2) is an enzyme involved in the biosynthesis of prostaglandins. Some studies have showed that it may be involved in liver cirrhosis [105]. In CCl4-treated mice, lncRNA-COX2 and COX2 levels are enhanced as compared to controls, and they are positively correlated with fibrosis development. These findings suggests that lncRNA-COX2 is involved in liver fibrosis development and that in the future it may be considered as a novel therapeutic target for treating liver fibrosis [106].

*Homebox transcript antisense RNA (HOTAIR)*

HOTAIR is an lncRNA located at the boundary of the homebox C (HOXC) locus on chromosome 12q13.13. It is implicated in different cellular functions and is increased in different cancer types. In CCl4-treated mice, the expression of HOTAIR was upregulated and HSCs were activated as compared to controls. Functional characterization of HOTAIR indicated that its overexpression activates fibrosis-related genes like matrix metalloproteinase 9 (MMP9) and MMP2, increases the levels of COL1A1 and actin alpha 2 (ACTA2), and promotes the cell proliferation. Moreover, HOTAIR can function as a regulator of the DNMT1/maternally expressed gene 3 (MEG3)/p53 pathway in HSCs [107]. Concerning NAFLD, HOTAIR upregulation determined by FAs increases TG accumulation in HepG2 cells and inhibits phosphatase and PTEN [108]. It has also been reported that HOTAIR is activated in NAFLD and that HOTAIR knockdown inhibits the development of NAFLD via regulation of the miR-130b-3p/Rho-associated coiled-coil containing protein kinase 1 (ROCK1)/AMPK axis [109]. HOTAIR can serve as a competing endogenous RNA to sponge miR-29b and then repress DNMT3b, both of which are involved in hepatic fibrosis development [110]. For patients infected with the hepatitis B virus, the increased HOTAIR expression levels indicate an accelerated evolution of fibrosis to HCC. HOTAIR holds oncogenic functions in HCC and it could have prognostic value. In the case of NAFLD, excessive circulating FFA levels determine an exaggerated upregulation of HOTAIR, which can be an important mechanism involved in liver steatosis. As a conclusion, HOTAIR may become a biomarker for liver injury [108].

*Nuclear enriched abundant transcript 1 (NEAT1)*

NEAT1 is a nuclear lncRNA involved in lipid uptake, lipolysis, and LDL oxidation. Consecutively, it is implicated in different hepatic diseases. NEAT1 knockdown causes a decrease in the migration, invasion, and proliferation of HCC cells via the regulation of nuclear ribonucleoprotein A2. NEAT1 plays a role in HSC activation. This has been demonstrated by the fact that NEAT1 overexpression increases the COL1A1 and ACTA2 levels and facilitates HSC activation [111]. In the livers and HSCs of CCl4-treated mice, NEAT1 expression improved, while knockdown of NEAT1 mitigated mouse fibrosis. In NAFLD and HCC animal models, the NEAT1 expression was upregulated [112].

Overexpression of NEAT1 decreases miR-122 levels, which in turn mediates the effects of NEAT1 on HSC activation, by a mechanism assigned to a Kruppel-like factor 6 (KLF6) [110]. In hepatocytes and cirrhotic liver tissues from subjects with unknown etiology, the NEAT1-miR-122-KLF6 axis operates to cause decreased miR-122 levels and increased KLF6 and NEAT1 levels. In HepG2 cells treated with FFAs and C57BL/6J mice treated with an HFD, NEAT1 and ROCK1 levels were found to be higher and miR-146a-5p levels were lower than those of controls. It was also demonstrated that NEAT1 and ROCK1 knockdown and miR-146a-5p overexpression mitigates lipid accumulation through AMPK pathway activation. So, NEAT1 can regulate NAFLD through miR-146a-5p, targeting ROCK1 [113]. Another study in an NAFLD rat model indicated an increase in NEAT1 expression and higher levels of FAS and ACC mRNAs. The knockdown of NEAT1 had a effect similar to the inactivation of the mammalian target of rapamycin (mTOR)/S6K1 pathway on the FAS and ACC mRNA levels. In rats, therefore, the downregulation of NEAT1 levels could improve NAFLD through the mTOR/S6K1-signaling pathway [114]. NEAT1 has a role in the activation of estrogen receptor α to regulate water-glycerol transporter (AQP7)-mediated hepatic steatosis [115].

In an NAFLD cellular model, NEAT1 knockdown mitigates fibrosis and inflammatory responses by regulating the miR-506/GLI3 axis [116]. For NASH model mice, it was found that NEAT1 and paternally expressed gene 3 were highly expressed in the HSCs and in the liver. Silencing NEAT1 significantly reduced the fibrotic characteristics of HSCs in NASH [117]. All of these findings confirm the fact that NEAT1 is upregulated in the fibrosis of NASH patients compared to controls and underlines the potential value of NEAT1 in NASH diagnosis and prognosis [118].

*Brown fat lncRNA 1 (Blnc1)*

Blnc1 can serve as a regulator of TGs biosynthesis due to its implication in the regulation of adipocyte function and differentiation. In mouse models of obesity and NAFLD, hepatic Blnc1 expression has been linked to lipogenesis activation. The knockdown of liver Blnc1 abrogated HFD-induced hepatic steatosis and insulin resistance and protected mice from diet-induced NASH pathogenesis [119].

In epididymal white fat tissue, the overexpression of Blnc1 partially mitigated glucose metabolism and dyslipidemia, improved insulin sensitivity, and protected against diet-induced obesity hepatic steatosis. These effects are probably due to the improvement of mitochondrial biogenesis and function in white fat [120]. The obtained results suggest that both in the liver and in white adipose tissues, Blnc1 has different regulatory mechanisms and functions.

*Apolipoprotein A4 Antisense (APOA4-AS)*

Apolipoprotein A4 is a plasma protein involved in the regulation of many metabolic pathways, including lipide and glucose metabolism. APOA4 is biosynthesized in the small intestine and in the hepatocytes and is then secreted into the blood. The mutations in APOA4 were correlated with an altered level of plasma lipid. APOA4 increases TG secretion and insulin production, inhibits gluconeogenesis, and is involved in obesity and type 2 diabetes pathology. APOA4-AS, as a reverse-transcript of the APOA4 gene, has been considered the regulatory lncRNA of APOA4. In vitro and in vivo studies concerning NAFLD have shown that APOA4-AS is essential to maintain APOA4 expression [121]. The knockdown of APOA4-AS in ob/ob mice hepatocytes causes a reduced level of APOA4, TC, and plasma TGs, which indicates the stabilizing role of APOA4-AS for APOA4. An RNA-binding protein named human antigen R is involved in the APOA4-AS mechanism of action. The human antigen R protein modulates mRNA stability and translation efficacy, which has an important role in growth, proliferation, and cell survival. In the APOA4-AS structure, there are two binding sites where human antigen R can bind. Studies have concluded that human antigen R is a stabilizing protein for APOA4-AS and APOA4. Human antigen is recruited to the APOA4-AS and APOA4 complex [122].

*Metastasis-associated lung adenocarcinoma transcript 1 (MALAT1)*

One of the most conserved lncRNAs is MALAT1. It is involved in diabetes, insulin resistance, and cancer. In primary HSCs from mice, knockdown of MALAT1 expression mitigates COL1A1 and ACTA2 levels and also diminishes the formation of the myofibroblast-like morphology specific to activated HSCs [71]. The inhibition of MALAT1 expression causes a decrease in the nuclear SREBP1c level and a decrease in lipid accumulation in vitro/in vivo. In turn, the increase of MALAT1 expression activates SREBP1c and causes lipid accumulation in hepatocytes. In a previous study, it was demonstrated that the presence of an excessive amount of palmitate is involved in increasing MALAT1 expression. In ob/ob mice, the insulin sensitivity of the liver was improved by MALAT1 reduction. So by increasing nuclear SREBP1c protein stability in the liver, MALAT1 can promote hepatic steatosis and insulin resistance [123]. After studying the liver biopsies of NAFLD patients, it was stated that MALAT1 acts as a regulator of hepatic inflammation and fibrosis and of insulin resistance by targeting the C-X-C motif chemokine ligand 5 (CXCL5). The inactivated liver LX-2 cells were enhanced by MALAT1 and CXCL5 expressions compared to control cells. These results suggest the role of modifying MALAT1 expression levels in hepatic fibrosis for NAFLD patients [118]. In another study, researchers compared NASH patients with NAFLD subjects with simple steatosis and controls. They found that MALAT1 expression was significantly increased only in NASH patients. MALAT1 is also involved in cells’ migration, proliferation, and invasion in different human cancers, including HCC. MALAT expression is increased in both HCC cell lines and liver tissue samples, highlighting its potential use as a biomarker of liver damage and HCC development [124].

*Maternally expressed gene 3 (MEG3)*

MEG3 is situated in the imprinted DLK1-MEG3 locus on human chromosome 14q32.3 region. It is also known under the name of “gene trap locus 2” and is involved in the progression of several types of cancers and as a regulator in carcinogenesis. It is also assumed to be involved in NAFLD pathogenesis [125]. MEG3 expression was decreased in the liver of CCl4-treated mice compared to the oil-fed control mice. This decrease is correlated with fibrosis progression. For fibrotic human patients, similar findings were reported. In in vitro and in vivo NAFLD models, MEG3 downregulation is negatively correlated with lipogenesis-related genes, and MEG3 overexpression mitigates excessive lipid accumulation in HepG2 cells. In two mouse NAFLD models (induced HFD and free fatty acid-challenged primary hepatocytes), MEG3 downregulation was found [126]. In HSC line and in LX-2 human cells, a dose- and time-dependent downregulation of MEG3 expression by transforming TGFβ1 was demonstrated. In contrast, in LX-2 cells, caspase-3-mediated apoptosis was promoted and TGFβ1-induced cell proliferation was inhibited by the upregulation of MEG3. In patients diagnosed with NASH, cirrhosis, and liver fibrosis, hepatic MEG3 levels were significantly increased [111]. In the vascular endothelium of diet-induced obese mice, MEG3 was one of the most differentially expressed lncRNAs. In NASH and human nonalcoholic fatty livers, MEG3 expression was elevated. MEG3 knockdown enhances obesity-induced insulin resistance and impaired glucose homeostasis [127]. The obtained results were contradictory, and they underlined the complexity of MEG3 regulation. Future studies are needed to validate MEG3’s biological importance and to establish whether MEG3 can be used as biomarker or a therapeutic target for NAFLD diagnosis/treatment [128].

*lncRNA Gm15622*

It has been demonstrated that Gm15622 upregulation increases lipid accumulation, while Gm15622 knockdown decreases lipid accumulation in an AML12 (alpha mouse liver 12) cell line. In the liver of high-fat diet obese, ob/ob, and db/db mice, Gm15622 was highly upregulated. Several studies have demonstrated that Gm15622 modulates SREBP-1c through miR-742-3p sponging. It was assumed that Gm15622 has a binding site for miR-742-3p. Because miR-742-3p was identified as a negative regulator of SREBP-1c and Gm15622 by sponging this miRNA, SREBP-1c protein enhancement is involved in NAFLD progression. It has been also shown that Gm15622 regulates the FAS enzyme via the siRNA-dependent knockdown of Gm15622 [129] and that metformin (a first-line medication for type-2 diabetes treatment) administration reduces SREBP-1c, Gm15622, and FAS expressions and increases miR-742-3p level. This is the reason why metformin is involved in NAFLD improvement/treatment [130].

*Highly upregulated in liver cancer (HULC)*

HULC is a functionally important lncRNA which is involved in HCC growth, metastasis, and also in drug resistance. It was the first identified lncRNA specifically overexpressed in HCC. HULC expression was found to be increased in the hepatic tissue of NAFLD rats. In the liver tissues of NAFLD rats, HULC inhibition improves lipid deposition and hepatic fibrosis and decreases hepatocyte apoptosis due to the inhibition of the mitogen-activated protein kinase (MAPK) signaling pathway [131]. In liver cancer cells, metformin administration decreased HULC by inhibiting the expression of specificity protein 1 (sp1), a transcription factor. It is known that metformin improves insulin resistance and increases insulin sensitivity and is recommended for NAFLD treatment [132]. After conducting these studies, it was concluded that HULC can become a target for NAFLD diagnosis, staging, and therapy.

As a conclusion, lncRNAs are known for their role as regulators of gene transcription and for their transcriptional regulatory functions. Many studies have demonstrated that lncRNAs play an important role in epigenetic and transcriptional regulation. LncRNAs are differently expressed in NAFLD patients than in healthy subjects. lncRNAs are also involved in NAFLD pathogenesis by different mechanisms. They are considered key players in liver metabolism regulation, mediators of inflammation, steatohepatitis, and fibrosis. In the last years, the role of lncRNAs in hepatic steatosis and in fatty liver has attracted more attention. Most studies related to lncRNAs and NAFLD have been carried out in vitro; a limited number of studies have been conducted in vivo. Due to the lack of conclusive deductions about the role of lncRNAs in NAFLD development in vivo, especially for NAFLD patients, the obtained results have not yet translated into clinical practice. The mechanisms of action of different lncRNAs on NAFLD and NASH development must be clarified, first on animal models and then on humans. Validation studies are also needed before the experimental results can be translated into clinical practice.

In liver diseases, different lncRNAs have potential importance in diagnostic, prognostic, and therapeutic protocols. Although it has been proven that lncRNAs can play an essential role in the mechanism of NAFLD, information concerning their involvement in the progression of the disease is scarce [71].

## 4. Circular RNAs and NAFLD

circRNAs represent a class of ncRNAs which contain miRNA response elements (MREs). They are non-linear RNAs that act as miRNA sponges and regulate gene expression. The first endogenous circRNA in humans was reported in 1991 [133]. circRNAs are single-stranded covalently closed RNA species formed through back-splicing, and many of them are located in the cell nuclei. The structure of circRNA is composed mostly of a circular loop RNA without a 5′-cap and 3′-tail [134]. Due to the fact that circRNAs contain multiple microRNA binding sites, they function as miRNA sponges involved in gene expression regulation. circRNA-miRNA-mRNA axes are involved in different signaling cascades in connection with apoptosis, vascularization, invasion, and metastasis. Circular RNAs have different potentially important functions, from miRNA and protein sponges to gene transcriptional regulators and protein/peptide translators. These are due to their specific characteristics like evolutionary conservation between species, exonuclease resistance, high stability, and existence in body fluids. At the transcriptional or post-transcriptional level, circRNAs can regulate pathogenicity-related gene expression [133].

**Figure 4:**
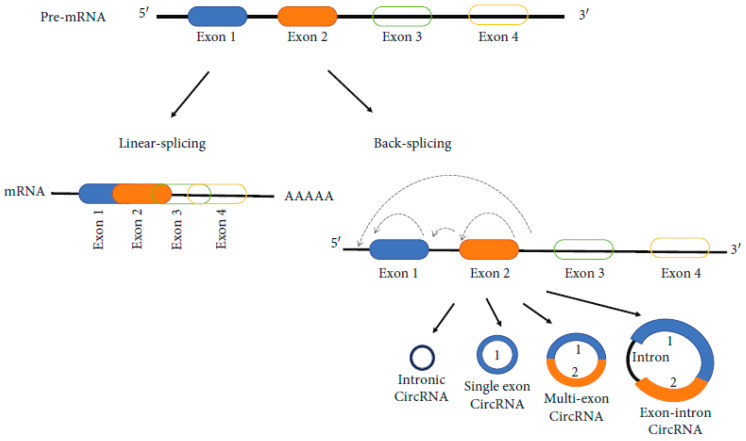
Biogenesis of circRNAs [71].

Due to the competition between the exonic linear splicing and a back-splicing circularization (an alternative splicing) which take place during the transcription of most human genes, the biogenesis of circRNAs occurs (Figure 4) [71].

Several subtypes of circRNA has been identified. Among these, four are primary. They are: (1) exonic circRNAs (ecircRNAs), derived primarily fromm single or several exons; (2) circular intronic RNAs (ciRNAs), which contain only introns; (3) exonic-intronic circRNAs (EIciRNAs), which contain both introns and exons; and (4) tRNA intronic circRNAs (tricRNAs) which are formed by splicing pre-tRNA introns. Most identified circRNAs are exonic circRNAs. ecircRNAs are primarily located in the cytoplasm. ciRNAs, eIciRNAs, and tricRNAs are primarily located in the nucleus, being involved in regulating parental gene transcription. Different mechanisms are involved in the production of circRNAs. CircRNAs are lncRNAs that undergo back-splicing and can come from transcripts containing both intronic and exonic fragments, only intronic fragments, or one or more exonic fragments [135].

Nowadays, many studies have proved the connection between circRNAs and the pathogenesis of metabolic diseases, but the utility of circRNAs in NAFLD is still under investigation [136]. Some studies have demonstrated the role of circRNAs in different essential processes involved in NAFLD onset and progression and that, in NAFLD, circRNAs display aberrant expression. In an NAFLD mouse model, it was reported that about 93 circRNAs were differentially expressed in the liver tissue, suggesting their role in liver steatosis [137]. In addition to its expression, the localization of circRNA may have a vital role in NAFLD progression and transition to NASH. Steatohepatitis-associated circRNA ATP5B regulator (SCAR), a mitochondrial circRNA, can participate in the activation of liver fibroblasts and in the enhancement of NASH-related fibrosis [138].

*circRNA_0046367 and circRNA_0046366/miR-34a/PPARα*

circRNA_0046367 and circRNA_0046366 act as endogenous regulators of miR-34a and are associated with NAFLD [113]. They block the interaction of miRNA/mRNA with MREs and can suppress the inhibitory impact of this latter on PPAR*α*. In pathological conditions, the PPAR*α* level increases, determining the activation of acyl-CoA-binding domain-containing 3 (ACBD3), CPT2, and of fatty acid transport protein SLC27A, and ultimately reducing steatosis. These findings indicate that the circRNA_0046366 or circRNA_0046366/miR-34a/PPAR pathways can represent epigenetic mechanisms underlying hepatic steatosis and may become a therapeutic alternative in NAFLD treatment [139].

*circRNA_0001805/miR-122/circPI4KB*

Primary human hepatocytes treated with FFAs exhibited reduced expression of circRNA_0001805 and showed increased susceptibility to inflammation [140]. However, transfection with circRNA_0001805 significantly attenuated hepatic inflammation, indicating a potential protective role for this circRNA in steatosis-induced inflammatory responses. More recently, circPI4KB has been identified as a regulator of miR-122 expression. It was shown that circPI4KB facilitates the export of miR-122 from hepatocytes to the extracellular space, leading to decreased intracellular levels of miR-122 and promoting lipid accumulation. These findings challenge the previously well established role of miR-122 as a key regulator of lipid metabolism within hepatocytes [141].

*circRNA_021412/miR-1972/LPIN1*

A relationship between miR-1972 and Lipin 1 (LPIN1) was highlighted in HepG2 cells treated with FAs. This confirmed the coregulation of LPIN1 expression by circRNA_021412 and miR-1972. LPIN1 determines the downregulation of long-chain acyl-CoA synthetases (ACSLs) expression and ultimately leads to steatosis development [142]. So, a decreased of circRNA_021412 levels can reduce the miR-1972 level and can inhibit LPIN1. As a conclusion, a circRNA-miR-mRNA signaling cascade seems to participate in hepatic steatosis regulation [139].

*circRNA_002581/miR-122/SLC1A5, PLP2, CPEB1*

The liver tissues of NASH mice were used to perform the profile of circRNAs expression, and it was found that 69 had an increased expression and 63 a reduced expression. A random selection of 13 from a total of 14 mRNAs and two from a total of 8 circRNAs was successfully validated by qRT-PCR. Four circRNA-miRNA-mRNA pathways were highlighted, including circRNA_002581-miR-122-Slc1a5, circRNA_002581-miR-122-Plp2, circRNA_002581-miR-122-Cpeb1, and circRNA_007585-miR-326-UCP2 [143]. These four genes are involved in NAFLD physiopathology. In mouse and cellular models of NASH, circRNA_002581 mitigates lipid droplet accumulation and improves hepatic damage, but the circRNA_002581-miR-122-CPEB1 axis aggravates NASH by autophagy suppression [144].

*circRNA_0067835/miR-155/FOXO3a*

Using LX-2 cells, a line of hepatic stellate cells (HSCs), which represent the main type of cells responsible for liver fibrosis, to identify thymosin beta 4 (T*β*4), a highly conserved 43 amino acid peptide that behaves as an antifibrotic and anti-inflammatory agent in vitro and in vivo, a microarray test was performed [145]. This study showed that circRNA_0067835, of the 644 differentially expressed circRNAs identified between control LX-2 cells and the T*β*4-depleted LX-2 cells, was significantly increased in the T*β*4-depleted LX-2 cells. A bioinformatics analysis assumed that circRNA_0067835 acts as a sponge of miR-155 to regulate the expression of Forkhead Box O3 (FOXO3a) [146].

*circRNA_0074410/miR-9-5p/KEGG pathway*

circRNA profiling of fibrotic HSCs revealed that 630 were downregulated and 179 were upregulated. Studies have demonstrated that circ_0074410 promotes HSCs activation and reduces miR-9-5p expression via the alpha smooth muscle actin (alpha-SMA) protein. Circ_0071410 expression knockdown mitigates hepatic stellate cells activation, which represents an important step in liver cirrhosis. The primary cell types responsible for liver fibrosis are also represented by HSCs. Hsa_circ_0071410 inhibition upregulates miR-9-5p expression, determining the attenuation of irradiation-induced HSC activation [147].

*circRNA_34116/miR-22-3p/BMP7*

In the CCl4-induced mouse model of liver fibrosis, 10,389 circRNAs were identified by microarray screening. Sixty-nine circRNAs were differentially expressed in the fibrotic liver tissues, of which 55 were downregulated and 14 were upregulated [148]. circRNA_34116 is one of the identified circRNAs. An in silico analysis assumed the presence of MRE of miR-22 on circRNA_34116 and showed that circRNA_34116 can competitively bind to miR-22-3p and indirectly regulate the transcription of its target gene bone morphogenetic protein 7 (BMP7) [149]. Networks between circRNAs and miRNA appeared as a new mechanism of gene expression regulation. They can help us to understand the molecular modulation of the disease development and progression and to dicover new therapeutic agents.

*circDIDO1/miR-143-3p*

The expression of circDIDO1 was found to be downregulated in HSCs following irradiation [147]. This observation prompted further investigation into its role in fibrosis-related processes. The results revealed that overexpression of circDIDO1 inhibited HSC proliferation and fibrogenesis by downregulating pro-fibrotic markers such as α-SMA and collagen I, promoting apoptosis and inducing cell cycle arrest. Additionally, the study identified the circDIDO1/miR-143-3p axis as a key regulator of the PTEN/AKT signaling pathway, underscoring its significance in modulating hepatic fibrosis [150].

*hsa_circ_0071410* and *circBNC2* have been shown to activate HSCs and hepatocytes, contributing to liver fibrosis in patients with NASH [147,151]. In contrast, *circFBXW4* exhibits an anti-fibrotic effect by acting as an HSC suppressor; it inhibits the fibrogenic pathway through its interaction with miR-18b [152].

In an in vitro model of NAFLD, *Hsa_circ_0048179* was shown to mitigate FFA-induced steatosis via *Hsa_circ_0048179/miR-188-3p/glutathione peroxidase 4* signaling. In a NASH mouse model, circRNA profiling identified *circRNA_29981* as the most differentially expressed circRNA [153].

Another research study suggested that *Exo-circCDK13* has the potential to alleviate liver fibrosis by suppressing the PI3K/AKT and NF-κB signaling pathways through the regulation of the *miR-17-5p/KAT2B* axis. This could directly impact the protein expression of collagen I and fibronectin [154].

In a mouse NAFLD model, *circ_0057558* promoted NAFLD by regulating ROCK1/AMPK signaling through targeting miR-206. In liver fibroblasts from patients with NASH, mitochondrial SCAR, a genome-encoded circRNA, was significantly downregulated [155]. SCAR overexpression also inhibited mitochondrial reactive oxygen species output and fibroblast activation via shutting down mitochondrial permeability transition pores. In vivo targeting of SCAR mitigates insulin resistance and HFD-induced cirrhosis. This means that *circRNA SCAR* can serve as a therapeutic target for NASH [156].

As a conclusion, the different localization of circRNAs determine their roles in the pathogenesis of NAFLD. This suggest that circRNAs’ expression and localization are involved in the occurrence and the NAFLD progression. Further studies are needed to elucidate precisely circRNAs’ functional mechanism in NAFLD development and their differences in NAFLD’s different stages, like NAFL, NASH, NASH-related fibrosis, NASH-related cirrhosis, and finally NASH-related HCC. The presented studies indicated that circRNAs are susceptible to contributing to the NAFLD phenotype, and they are considered to be possible targets for NAFLD diagnosis and therapy. CircRNA and lncRNA have cell-type and tissue-specific expression patterns, and they can be released in different body fluids, like blood and urine, where they have a good stability. Therefore, their utility as new biomarkers of NAFLD promises great advantages and may have a great impact in clinical practice.

## 5. Piwi-Interacting RNAs and NAFLD

PiRNAs are small ncRNAs that interact with Piwi-Argonaute proteins in the germ line. They are named after their biological function. In addition to their established role in spermatogenesis, piRNAs also function as key regulators in transposon silencing [157]. However, their involvement in metabolic diseases remains largely unexplored, and to date only a few studies have investigated their potential role in NAFLD.

Using microarray technology, differential expression of piRNAs was identified in liver tissues from an NAFLD mouse model [158]. The findings suggest that piRNAs may play a regulatory role in NAFLD by interacting with proteins involved in various metabolic pathways. In silico analysis further revealed that dysregulated piRNAs are associated with key cellular components in liver tissue, indicating a potential role in liver cell activation.

The relationship between piR-823 and HSC activation has been investigated through loss and gain of function assays using a piR-823 antagomir and piR-823 mimic. Modulating piR-823 levels significantly influenced HSC behavior; overexpression of piR-823 promoted HSC proliferation, whereas its inhibition increased the proportion of quiescent cells by targeting the TGF-β pathway [159]. These findings suggest that piR-823 plays a critical role in HSC activation and may contribute to the development of liver fibrosis. It is well established that macrophages, including Kupffer cells, play a central role in initiating, maintaining, or resolving inflammation within tissues. Recent studies have shown that various ncRNAs, including piRNAs, are secreted by macrophages enclosed within extracellular vesicles. These ncRNAs are believed to influence the immune response in the tissue microenvironment by modulating the activity of other cell types, thereby either promoting or suppressing inflammation. However, due to limitations in current piRNA annotation and analysis pipelines, misinterpretation of data remains a concern and may impact the accuracy of study conclusions [160,161].

As a conclusion, piRNAs represent a promising underexplored area in NAFLD research. Nowadays, technology is constantly advancing, so detecting and studying piRNAs in blood and liver tissue may emerge as key players in liver metabolism, immune response, and disease progression from NAFLD to NASH and finally to HCC. Interactions of piRNAs with other ncRNAs, like piRNA-lncRNA or piRNA-miRNA, are also studied. These interactions may elucidate how piRNAs influence gene expression beyond transposon silencing, and how they coordinate with miRNAs and lncRNAto control the complex pathogenesis involved in NAFLD.

## 6. The Role of ncRNA in NAFLD—Associated Metabolic Dysfunction

The importance of miRNA, lncRNA, circRNA, and piRNA in NAFLD lies in their different roles, mechanisms of action, and regulatory targets. While they all contribute to the development and progression of NAFLD, their functions differ in how they influence gene expression, lipid metabolism, inflammation, and fibrosis.

A few years ago, the name NAFLD become metabolic associated non alcoholic fatty liver disease (MAFLD). The renaming is due to the pathological factors involved in the fatty liver development, and it is useful in the elucidation of the disease progression and in MAFLD management. MAFLD reflects the role of metabolic factors involved in liver steatosis. Metabolic syndromes (diabetic, obese, hyperlipidemia patients) are considered as risk factors in NAFLD development. The presence of one or more metabolic risk factors determines insulin resistance, an increase of lipolysis, and an overall systemic disturbance. The existence of a large amount of FFAs in the liver determines triglyceride synthesis, reduces FFAs catabolism, and enhances fat deposition in the liver.

Obesity is closely related to impaired blood glucose tolerance and with hyperinsulinemia, which increases the risk of developing diabetes and hyperlipidemia [162]. Obese patients might or might not be diagnosed with metabolic syndrome, but this does not exclude the presence of a fatty liver [163]. This contradiction is not fully understood, but it is stated that the impaired insulin signaling seen in patients with a preexistent metabolic condition represents the main element in NAFLD progression.

Many studies have indicated the role of ncRNAs in metabolic modulation involving insulin signaling, namely that they prevent or contribute to metabolic abnormalities within hepatic cells. Some miRNAs were found to be deregulated in obese animal models as well as in human hepatic tissue and were found to regulate both glucose and lipid homeostasis. lncRNAs were also linked to impaired metabolic signaling related to obesity. The role of lncRNAs in response to insulin is still not elucidated. The data suggest that metabolic regulation might be controlled by a single or a group of lncRNA and they could have a role in the pathogenesis of insulin-related pathologies. Concerning circRNAs, deep sequencing analysis followed by gain- and loss-of-function approaches found that they are protective in metabolic syndrome patients [164].

The use of ceRNA networks (competing endogenous RNA networks) in NAFLD research is a rapidly growing area that aims to uncover the complex regulatory interactions between different types of noncoding RNAs and how these interactions influence gene expression and disease progression. It provides a layer of post-transcriptional regulation in NAFLD beyond the classic pathways (insulin resistance, oxidative stress, lipid accumulation) and it can help to elucidate how ncRNAs contribute to NAFLD pathogenesis. Many ceRNA networks remain at the level of bioinformatics/computational predictions, fewer are confirmed in vivo (on animal) or in patient samples [165].

NAFLD screening recommendations are applied to patients with preexisting metabolic disease, in diabetic or obese patients, and to patients with hyperlipidemia. Investigation of metabolic factors and ncRNAs roles in NAFLD development must be carried out by an holistic approach [162].

Alterations in miRNA, circRNA, and lncRNA expression have an important role in cellular physiology in NAFLD and HCC. The different changes in the expression levels of ncRNAs along with their target effectors can underline the importance of ncRNAs in the different stages of NAFLD progression. Patients with NAFLD show distinct profiles of ncRNAs. The changes in the expression and/or localization of ncRNAs function as liver activators can contribute to the progression of NAFLD into NASH. Dysregulated ncRNAs were shown to upregulate lipotoxicity, oxidative stress fibrosis, inflammation, and metabolic dysfunction in NAFLD [164]. To improve steatosis and fibrosis, the patient’s lifesyle must be improved and weight loss must be achieved by following a healthy diet. Nowadays, treatment options include drugs which only improve disease progression and control NAFLD/NASH symptoms. These are drugs belonging to the antidiabetic, PPAR agonist, FXR agonist, and THR agonist classes [1].

Obtaining more information about the NAFLD-regulated ncRNAs, and in-depth understanding of their roles in the disease will help to develop a highly effective targeted therapy. Understanding the regulatory pathways involving ncRNA in NAFLD initiation and progression is challenging, because other factors are implicated in the disease. To date, no ncRNA has been introduced into clinical practice as a novel drug for NASH/NAFLD treatment.

Some ncRNAs can be expressed inconsistently or even inversely in different tissue compartments. For example, they can be dysregulated in intrahepatic samples and upregulated in serum, suggesting that ncRNAs can play different roles related to the biological or disease context. This intracellular–extracellular expression was highlighted during NAFLD progression [166]. In this context, periodic dynamic measurements of tissue and circulating ncRNA expression to track disease changes in real-time are needed. Most of the studies determined the functional activity and diagnostic performance of a single ncRNA, frequently failing to distinguish between NAFLD, NASH, and fibrosis. We recommend further exploration of the co-regulatory networks and combined effects of these ncRNAs. As described above, some ncRNAs can have different or even opposite roles in different liver diseases, and so cell- and disease-specific ncRNAs may present great advantages in discovering biomarkers for NAFLD. Future research must focus on cell- and disease-specific ncRNAs and on the combined effect of various ncRNAs on multiple targets, which will lead to the discovery of new and valuable biomarkers for NAFLD diagnosis [156].

As a conclusion, ncRNAs represent promising non-invasive biomarkers for the diagnosis and stratification of patients with NAFLD and could help to develop personalized treatments for NAFLD. However, the research in the field has met some limitations and technical problems. Unfortunately, most of the studies were performed on patients who had worsening symptoms and for this reason needed medical assistance. The status of ncRNAs must be investigated in the general population, aiming to diagnose the disease in its early stages when the patient is asymptomatic and to determine if the ncRNA overexpression is the trigger or it is just a consequence of other causes such as those associated with lipid metabolism disorder, inflammation, or immune system disorders [124].

The role of ncRNAs as mediators of cell and organ crosstalk as well as their impact on different signaling pathways involved in NAFLD pathogenesis are not fully understood. Special attention should be drawn to further research addressing the role of ncRNAs and their carriers (extracellular vesicles) in mediating potential inter-organ crosstalk in NAFLD condition, and the dynamic interaction of ncRNAs with metabolism cell signaling pathways. These strategies can be a starting point for increasing our knowledge on the role of circulating ncRNAs in organ crosstalk and may represent an opportunity to better understand how they affect metabolic homeostasis to drive the onset and progression of NAFLD and related pathological conditions [166].

It is difficult to diagnose a multifactorial disease such as NAFLD using a single ncRNA. Attempts have been made to use a combination of serum circulating ncRNAs. Understanding the ncRNA-ncRNA crosstalk and their intricate interplay with different genetic and other epigenetic regulators, including DNA methylation, chromatin remodeling, and components of the transcriptional and post-transcriptional machineries to regulate gene networks involved in NAFLD, could certainly expand the knowledge on the molecular mechanisms driving this disease [124].

## 7. Clinical Utility of Noncoding RNAs in NAFLD

An increasing number of studies support the role of miRNAs in the pathogenesis of NAFLD. These molecules are not only essential for elucidating the underlying mechanisms of disease progression, but also hold significant promise as biomarkers for diagnosing and staging NAFLD, as well as therapeutic targets for halting or reversing its advancement [167]. Nevertheless, several challenges in this field still need to be addressed.

### 7.1. miRNAs as NAFLD Biomakers

Extracellular miRNAs have emerged as valuable biomarkers for various human diseases, owing to their distinct expression patterns that reflect the cellular origin and specific physiological or pathological conditions. In particular, miRNA profiling from blood samples has been extensively studied for its potential to enhance the diagnosis of NAFLD and NASH. Several recent meta-analyses have assessed the current state of research in this area. Circulating miRNA expression profiles have been investigated across different stages of human NAFLD, revealing that elevated levels of miR-122, miR-34a, and miR-192 may help distinguish NASH from NAFL. However, sufficient data were only available to assess the diagnostic accuracy of miR-122 and miR-34a, both of which demonstrated only moderate performance in differentiating NAFLD from healthy controls and NASH from NAFL, respectively [168]. There were too few studies available to determine which specific miRNAs could effectively differentiate fibrosis stages in NASH. A more recent systematic review and meta-analysis investigated the potential of serum miRNA profiles to diagnose total NAFLD, NAFL, and NASH. This analysis focused on the diagnostic performance of three miRNAs, miR-34a, miR-122, and miR-99a, as serological biomarkers for NAFLD. The findings indicated that, overall, serum miRNAs demonstrated greater diagnostic accuracy for NASH than for total NAFLD, with particularly high efficacy in distinguishing NASH from NAFL. Although all three miRNAs showed moderate performance in diagnosing NAFLD, miR-34a exhibited the lowest heterogeneity and the most consistent diagnostic performance. However, its individual diagnostic efficacy for NASH was not assessed separately [169].

Reduced plasma levels of miR-122 have been identified as a risk factor for mortality in individuals with NAFLD. A recent study evaluated the associations between circulating levels of miR-34a, miR-122, miR-192, and miR-200a and both histological features and pathogenic mechanisms of NAFLD. MiR-34a, miR-122, and miR-192 were independently linked to hepatic steatosis, as well as to key pathogenic factors such as insulin resistance and established NAFLD-associated genetic polymorphisms. MiR-200a, in contrast, was associated with only one genetic risk variant. All four miRNAs demonstrated a strong, positive correlation with advancing stages of liver fibrosis. The predictive value of miR-34a alone, as well as the combination of all four miRNAs, was compared with that of FIB-4 (a validated score for liver fibrosis). While miR-34a and the miRNA panel showed similar sensitivity and specificity to FIB-4 in predicting advanced fibrosis (stage 3 or higher), and slightly lower performance for stage 4, they outperformed FIB-4 in detecting early-stage fibrosis. However, their overall diagnostic accuracy remained moderate. Therefore, although miR-34a, miR-122, and miR-192 are currently among the most promising candidates for non-invasive biomarkers of NAFLD progression, their combined diagnostic power remains limited, particularly in distinguishing between different types of liver disease or specific NAFLD stages. This conclusion aligns with findings from a recent review evaluating the role of circulating miRNAs as biomarkers in human NAFLD [61].

The development and application of techniques to selectively isolate circulating extracellular vesicles (EVs) of hepatic origin may facilitate the identification of miRNA biomarker panels for more accurate diagnosis and staging of NAFLD. In fact, a method for the specific isolation of hepatocyte-derived EVs from human plasma has recently been established [170]. As an initial step toward applying this method for NAFLD diagnostics, hepatocyte-derived extracellular vesicles (EVs) were isolated from plasma samples of healthy individuals, as well as those with NAFL or NASH, followed by extraction of miRNAs. Levels of three selected miRNAs, miR-122, miR-192, and miR-128, were significantly elevated in hepatocyte-derived EVs from individuals with NASH compared to healthy controls. Furthermore, these miRNA levels showed a positive correlation with disease severity and demonstrated superior diagnostic performance in distinguishing NAFLD from controls, compared to the same miRNAs extracted from total plasma cell-free RNA or from globally derived plasma EVs [171]. However, the method employed is limited to isolate only EVs of hepatocyte origin. In addition, only three selected miRNAs were analyzed in a small cohort of individuals (n = 6–14), with minimal accompanying clinical data. Advancing techniques to selectively capture EVs derived from other cell and tissue types implicated in NAFLD pathogenesis, combined with comprehensive, miRNome-wide screening to identify the most informative biomarker candidates, may lead to the development of a miRNA panel with high diagnostic accuracy, thereby enhancing the effectiveness of NAFLD diagnostics. The diagnostic algorithm NIS4™ enhances the diagnostic and prognostic utility of circulating miRNAs in NAFLD by integrating their quantification with that of additional circulating biomarkers. Specifically, NIS4 quantifies a panel of four blood-based markers: miR-34a, two fibrosis-related proteins (alpha-2-macroglobulin and chitinase-3-like protein 1), and hemoglobin A1c, a marker of long-term glycaemic control. In a large pooled validation cohort comprising over 700 patients, NIS4 was evaluated for its ability to identify individuals with NASH who are at high risk for severe long-term liver outcomes, particularly among those with metabolic risk factors. The algorithm significantly outperformed five out of six currently used non-invasive clinical tests and reliably identified at-risk individuals regardless of age, sex, or body mass index (BMI). These findings position NIS4 as a promising alternative to liver biopsy for stratifying at-risk patients across the full spectrum of NASH and fibrosis [172]. Another potential clinical application of circulating miRNAs is their integration with standard non-invasive diagnostic methods, whereby quantification of plasma-derived miRNAs could enhance the accuracy of NAFLD/NASH diagnosis and fibrosis staging [168].

### 7.2. miRNAs as Targets of NASH Pharmacotherapies

miRNAs represent promising pharmacotherapeutic targets because of their ability to regulate multiple downstream pathways in a manner that is both cell- and tissue-specific. In response to this potential, several pharmaceutical companies are actively developing miRNA-based therapies aimed at treating various human diseases. These approaches typically involve chemically stabilized oligonucleotides, which may function either as mimics to restore the activity of specific miRNAs downregulated in disease, or as AntagomiRs designed to inhibit the function of miRNAs that are overexpressed or implicated in disease pathogenesis. A range of delivery strategies is being explored to ensure the targeted delivery of these therapeutics to specific cell types [173]. Conjugation with N-acetylgalactosamine (GalNAc), a ligand for the asialoglycoprotein receptor, is a commonly employed strategy for achieving hepatocyte-specific delivery of oligonucleotides [174]. Alternative delivery systems have been explored in non-clinical in vitro and in vivo studies to selectively target oligonucleotides to HSCs or Kupffer cells [175]. In the future, these approaches may enable the targeted delivery of therapeutic oligonucleotides designed to mimic or inhibit miRNAs implicated in NASH and/or fibrosis, offering potential treatment strategies for more advanced stages of NAFLD.

### 7.3. lncRNAs and circRNAs as Biomakers and Therapeutic Potential in NAFLD

Gain- and loss-of-function strategies involving lncRNAs and circRNAs have demonstrated significant influence on the pathophysiology and phenotype of NAFLD, highlighting their potential as therapeutic targets. These noncoding RNAs typically exhibit tissue-specific expression patterns and are expressed at lower levels than mRNAs, allowing for therapeutic administration at reduced doses with potentially fewer toxic side effects. However, the functional relevance of their low expression levels remains a subject of debate, underscoring the importance of identifying key functional domains within these ncRNAs that mediate specific interactions with other RNAs, DNA, or proteins. Current targeting technologies like expression vectors, delivery platforms, antisense oligonucleotides, and CRISPR-Cas9/13 systems show considerable promise. Nevertheless, their clinical application requires further refinement to address safety concerns and minimize off-target effects [176]. Additionally, lncRNAs are generally longer than circRNAs, which can lead to the activation of the immune system and pose greater challenges for efficient delivery. The limited sequence conservation of lncRNAs across species further complicates their clinical translation and applicability to human diseases. However, despite sequence variability, many lncRNAs retain conserved secondary or tertiary structures, which may preserve their functional roles across species [177,178]. Thus, a comprehensive understanding of lncRNA functional structures is essential for the rational development of lncRNA-targeted therapeutics. Concerning circRNA underlying mechanisms of action, studies are still needed. circRNA presents a great advantage, namely its high stability in the circulation. This recommends circRNAs as potential biomarkers or therapeutic agents in NAFLD pathology [179]. However, nowadays, new studies are needed to validate the use of circRNAs as NAFLD biomarkers.

## 8. Future Perspectives

NAFLD progression varies considerably among individuals, with gene expression patterns during disease development being influenced by factors such as genetic background, diet, and the presence of metabolic comorbidities [180]. Similarly, animal models used to investigate the role of miRNAs in NAFLD pathogenesis differ in genetic makeup, dietary protocols, and methods of disease induction, resulting in variable similarity to human NAFLD/NASH [181]. These differences likely contribute to the inconsistencies observed across experimental studies examining the role of specific miRNAs, such as miR-21 or miR-122, in NAFLD development. This highlights the need for future research utilizing carefully controlled experimental models that more accurately replicate specific stages of human NAFLD. When using rodent models to explore the functional roles of particular miRNAs in human NAFLD, it is also critical to consider the evolutionary conservation of those miRNA species between the model and humans. Crucially, the relevance of an miRNA in a given context depends on whether its interaction with target genes is conserved in the specific cellular environment being studied. Moreover, functional redundancy of a specific miRNA-target interaction may exist in the experimental model organism, meaning that additional miRNA species may need to be depleted before any effects of miRNA inhibition can be observed [182].

Most studies addressing the involvement of miRNAs in NAFLD pathogenesis have focused on the downstream effects of miRNA actions rather than the upstream regulatory mechanisms responsible for the gain or loss of miRNA expression during disease progression. A key objective for future research will be to deepen our understanding of the complex gene regulatory networks involved in NAFLD by elucidating the upstream factors responsible for their dysregulation. This includes identifying critical transcription factors that drive widespread changes in miRNA expression and their downstream protein targets across well-defined early, intermediate, and late stages of NAFLD.

Exploring the largely uncharted area of cis-regulatory DNA elements, specifically enhancers that transcriptionally regulate miRNA expression in human NAFLD, will represent a key frontier in future research. miRNAs are thought to contribute to NAFLD pathogenesis not only through their roles as endogenous regulators of gene expression within hepatocytes, HSCs, or Kupffer cells, but also by facilitating intercellular communication in the liver following their release [183]. Future research should aim to distinguish between circulating miRNAs that function solely as biomarkers of organ-specific dysfunction in NAFLD and those that play a mechanistic role in mediating the intercellular and inter-organ communication that drives disease progression. While miRNAs used as diagnostic biomarkers may be clinically valuable for disease staging and identifying patients at increased risk of progression, those miRNAs (and also their upstream regulators, many of which remain to be identified) that are directly involved in the pathogenesis of NAFLD may represent promising therapeutic targets for NASH. Given that cardiovascular disease remains the leading cause of mortality in individuals with NAFLD, including those with NASH [182], the development of therapies that address not only liver-specific molecular pathways but also those contributing to cardiovascular dysfunction would offer substantial clinical benefits. Identifying miRNAs that mediate communication between these two organ systems, along with elucidating the regulatory mechanisms controlling their expression and downstream effects, may represent a critical step toward achieving this goal.

The integration of computational methods, such as bioinformatics and biomodeling, with high-throughput genomic sequencing technologies holds significant promise for uncovering novel gene regulatory networks and predicting miRNA targets. When combined with advanced quantitative proteomic techniques, these approaches may mark the beginning of a new era in the discovery of genes involved in NAFLD and cancer, potentially offering new strategies for their diagnosis and treatment. Emerging systems biology approaches, which provide a holistic view of complex biological processes and disease pathways, are expected to play a pivotal role in clarifying the mechanisms underlying NAFLD pathogenesis, thereby greatly enhancing efforts in early diagnosis and prevention [20]. Overall, the dual role of miRNAs, both as contributors to pathophysiological processes across multiple organs and as promising biomarkers for hepatic disease screening, must be carefully evaluated and investigated to enhance diagnostic accuracy in patients. Consequently, further research addressing these aspects is essential before miRNA-based strategies can be fully validated in clinical trials. Nonetheless, current evidence suggests that miRNAs hold considerable potential as valuable tools for clinicians in improving the diagnosis and prognosis of NAFLD. 

Current evidence suggests that functionally significant lncRNAs and circRNAs play key regulatory roles in the molecular pathways underlying NAFLD and show considerable potential as biomarkers for disease risk assessment, diagnosis, treatment, and prognosis. However, most findings to date are limited to preclinical models and require validation in larger, independent human cohorts. Further research is also needed to address several challenges, including the typically low expression levels of these noncoding RNAs, limitations in detection methodologies, the complexity of identifying functional domains, and the diverse pathophysiological features of liver disease. Given their cell- and tissue-specific expression patterns, advancing lncRNA and circRNA profiling at the single-cell level could significantly enhance biomarker discovery. Additionally, spatial transcriptomics offers a promising approach to studying RNA expression within the complex architecture of liver tissue [156].

Concerning NAFLD treatment, there is the possibility to target lncRNAs for therapeutic use. The inhibition or mimicking of a single or a set of lncRNAs represents one of the promising approaches in targeted NAFLD therapy. The inhibition approaches can be used to knockdown upregulated lncRNAs with the aim of pathological process prevention. In turn, mimicking can be used to re-express the downregulated lncRNA. The use of these inhibitors/stimulators may become promising for treating NASH and NAFLD. For the purpose of using lncRNAs as biomarkers for NAFLD, for lncRNA detection and identification in biofluids and hepatic tissues, the techniques used for their detection and identification must be improved. This represents another challenge for the future [130].

NAFLD development involves numerous genetic and epigenetic factors. In the future, certainly, new genetic determinants of NAFLD will be discovered. To fully elucidate the mechanism underlying the pathogenesis of NAFLD, the interaction between the inherited risk factors and the epigenetic changes will require increased attention and more investigations. Today, weight loss represents the only therapy that gives relative results in NAFLD treatment. To this end, lifestyle modifications are required. To assess and monitor the severity of NAFLD by circulating ncRNA determination, more studies are needed to identify and validate the efficiency and accuracy of these markers and to determine their therapeutic potential. As a conclusion, studying the ncRNAs in NAFLD could elucidate the pathophysiology of the disease, allowing them be used as predictive, diagnostic, and prognostic biomarkers for NAFLD, and aid the discovery of a new drug for NAFLD treatment.

## 9. Conclusions

As metabolic diseases are on the rise, and steatosis in particular is becoming more prevalent, early detection of its progression to a disease state (NAFLD) is necessary. We consider that the current article addresses a very relevant and current topic. The novelty and distinct contributions of the current review consist in presenting most of the ncRNAs (miRNA, lncRNA, circRNA, and piRNA) involved in the pathogenesis of NAFLD, following known aspects related to in vitro and in vivo studies (on animals/humans). Considering the desire to use ncRNA for diagnostic and therapeutic purposes, aspects regarding future research were also addressed.

## Data Availability

No new data were created or analyzed in this study. Data sharing is not applicable.

## Abbreviations

ABCA1/ABCG1	ATP-binding cassette transporters A1/G1
ACBD3	acyl-CoA-binding domain-containing 3
ACC/ACC1	acetyl-CoA carboxylase /acetyl-CoA carboxylase 1
ACLY	ATP citrate lyase
ACSLs	long-chain acyl-CoA synthetases
ACTA2	actin alpha 2
alpha-SMA	alpha smooth muscle actin
AMPK	AMP-activated protein kinase
APOA4-AS	apolipoprotein A4 Antisense
ApoB	apolipoprotein B
APTR	alu-mediated transcriptional regulator
ARNTL	aryl hydrocarbon receptor nuclear translocator-like 1 protein
Blnc1	brown fat lncRNA1
BMI	body mass index
BMP7	bone morphogenetic protein 7
CD36	cluster of differentiation 36
ceRNAs	competitive endogenous RNAs
circRNAs	circular RNAs
ciRNAs	circular intronic RNAs
ChREBP	carbohydrate-responsive element-binding protein
CoA	coenzyme A
COL1A1	type 1 collagen chain
COX2	cyclooxygenase 2
CPT1A/CPT2	carnitine palmitoyltransferase 1A/carnitine palmitoyltransferase 2
CRISPR	clustered regularly interspaced short palindromic repeats
CSCs	cancer stem cells
CXCL5	C-X-C motif chemokine ligand 5
DLK1	delta-like non-canonical Notch ligand 1
DNMT3β/DNMT1	DNA methyltransferase 3β/1
EBP	enhancer binding protein
ecircRNAs	exonic circRNAs
EIciRNAs	exonic-intronic circRNAs
eIF2a	eukaryotic initiation Factor 2a
EpCAM	epithelial cell adhesion molecule
ER	endoplasmic reticulum
ERK	extracellular signal-regulated kinase
EVs	extracellular vesicles
FAs	fatty acids
FAS	fatty acid synthase
FFA	free fatty acids
FLRL2	fatty liver-related lncRNA 2
FOXO3a	forkhead Box O3
FXR	farnesoid X-activated receptor
GATA	GATA-binding protein 4
H19	lncRNA H19
HCC	hepatocellular carcinoma
HDL	high-density lipoprotein
HFD	high-fat diet
HMGCR	3-hydroxy-3-methylglutaryl-co-enzyme A reductase
HMOX1	heme oxygenase 1
HNF4-α	hepatocyte nuclear factor 4-alpha
HOTAIR	homebox transcript antisense RNA
HOXC	homebox C
HSCs	hepatic stellate cells
HULC	highly upregulated in liver cancer
HUVEC	human umbilical vein endothelial cells
IL6	interleukin 6
INK4A	tumor suppressor gene p16
IRG 1	immuno-responsive gene 1
JNK	janus kinase
KLF6	kruppel-like factor 6
LDL	low-density lipoprotein
lncARSR	lncRNA activated in RCC with sunitinib resistance
lncHR1	lncRNA HCV regulated 1
lncRNAs	long noncoding RNAs
LPIN1	Lipin 1
LXR	liver X receptor
MALAT1	metastasis-associated lung adenocarcinoma transcript 1
MAFLD	metabolic associated non alcoholic fatty liver disease
MAPK	mitogen-activated protein kinase
MCD	methionine-choline deficient
MEG3	maternally expressed gene 3
miRNAs	microRNAs
MMP9/MMP2	matrix metalloproteinase 9/2
MREs	miRNA response elements
mTOR	mammalian target of rapamycin
MTTP	microsomal triglyceride transfer protein
NAFL	nonalcoholic fatty liver
NAFLD	nonalcoholic fatty liver disease
NASH	nonalcoholic steatohepatitis
ncRNAs	noncoding RNAs
NEAT1	nuclear enriched abundant transcript 1
NF-κB	nuclear factor kappa B
NRF 1	regulator nuclear respiratory factor 1
NRF2	nuclear factor erythroid 2-related factor 2
PECAM1	platelet-endothelial cell adhesion molecule-1
PERK	PKR-like ER Kinase
PGC1b	PPAR gamma coactivator 1-b
PI3K	phosphoinositide 3-kinases
piRNAs	piwi-interacting RNAs
PPARα/PPARγ	peroxisome proliferator-activated receptor alpha/gamma
PTEN	phosphatase and tensin homolog
RCC	renal cell carcinoma
RIPK1	receptor-interacting protein kinase 1
ROCK1	Rho-associated coiled-coil containing protein kinase 1
RTPCR	real-time polymerase chain reaction
RUNX1	runt-related transcription factor 1
RXRα	retinoid X receptor α
SCAR	steatohepatitis-associated circRNA ATP5B regulator
SCD1	stearoyl-CoA desaturase 1
SIRT1	sirtuin 1
SMAD	subsequently phosphorylating receptor-activated
sp1	specificity protein 1
SREBP	sterol regulatory element-binding protein
T*β*4	thymosin beta 4
TC	total cholesterol
TG	triglycerides
TGF-*β*1	transforming growth factor-*β*1
TISCs	tumor-initiating stem cells
TNF	tumor necrosis factor
tricRNAs	tRNA intronic circRNAs
UC372	ultra-conserved element
VCAM1	vascular cell adhesion molecule-1
VEGF	vascular endothelial growth factor
VLDL	very low-density lipoprotein
Wnt	wingless
XBP1	X-box binding protein
YAP1	yes-associated protein 1
